# Inhaled corticosteroids and adverse outcomes among chronic obstructive pulmonary disease patients with community-acquired pneumonia: a population-based cohort study

**DOI:** 10.3389/fmed.2023.1184888

**Published:** 2023-07-24

**Authors:** Damien Basille, Lei Wang, Reimar Wernich Thomsen, Jyothi Menon, Nisha Shetty, Pierre Duhaut, Claire Andrejak, Vincent Jounieaux, Henrik Toft Sørensen

**Affiliations:** ^1^Department of Clinical Epidemiology, Aarhus University Hospital and Aarhus University, Aarhus, Denmark; ^2^Department of Respiratory Disease and Critical Care Unit, University Hospital Centre Amiens-Picardie, Amiens, France; ^3^AGIR Unit–UR4294, University Picardie Jules Verne, Amiens, France; ^4^RECIF Unit, University Picardie Jules Verne, Amiens, France; ^5^Department of Internal Medicine, University Hospital Centre Amiens-Picardie, Amiens, France

**Keywords:** cohort study, community-acquired pneumonia, inhaled corticosteroids, mortality, pleural empyema

## Abstract

**Introduction:**

While inhaled corticosteroids (ICS) may increase pneumonia risk in patients with chronic obstructive pulmonary disease (COPD), the impact of ICS on pneumonia outcomes is debated. We examined whether ICS use is associated with adverse outcomes among COPD patients with community-acquired pneumonia (CAP).

**Materials and methods:**

Population-based cohort study of all COPD patients with an incident hospitalization for CAP between 1997 and 2013 in Northern Denmark. Information on medications, COPD severity, comorbidities, complications, and death was obtained from medical databases. Adjusted risk ratios (aRRs) for pleuropulmonary complications, intensive care unit (ICU) admissions, and 30-day mortality in current and former ICS users were compared with those in non-users, using regression analyzes to handle confounding.

**Results:**

Of 11,368 COPD patients with CAP, 6,073 (53.4%) were current ICS users and 1,733 (15.2%) were former users. Current users had a non-significantly decreased risk of pleuropulmonary complications [2.6%; aRR = 0.82 (0.59–1.12)] compared to non-users (3.2%). This was also observed among former users [2.5%; aRR = 0.77 (0.53–1.12)]. Similarly, decreased risks of ICU admission were observed among current users [aRR = 0.77 (0.57–1.04)] and among former users [aRR = 0.81 (0.58–1.13)]. Current ICS users had significantly decreased 30-day mortality [9.1%; aRR = 0.72 (0.62–0.85)] compared to non-users (12.6%), with a stronger association observed among patients with frequent exacerbations [0.58 (0.39–0.86)]. No significant association was observed among former ICS users [0.89 (0.75–1.05)].

**Conclusion:**

Our results suggest a decreased risk of death with ICS use among COPD patients admitted for CAP.

## Introduction

Chronic obstructive pulmonary disease (COPD) is a strong predictor of hospitalization for community-acquired pneumonia (CAP). In a cohort study of patients aged 40 years and older, the annual incidence of hospitalization for CAP was 9.4% in patients with COPD versus 0.5% in patients without COPD (1). The increased risk of pneumonia in COPD patients seems partly due to impaired lung defenses (2–4), but may also relate to use of inhaled corticosteroids (ICS) as shown by observational studies and meta-analyzes of randomized controlled trials (RCTs) (5–10).

Existing data on the association between ICS and outcomes in patients with pneumonia are conflicting. Sellares et al. reported that both COPD and non-COPD patients who used ICS before CAP had a lower incidence of parapneumonic effusion (4). Whether ICS use impacts mortality in COPD patients with pneumonia also remains debated (9). RCTs reported no difference in pneumonia-associated or all-cause mortality between ICS and non-ICS users, but the number of outcomes were few hampering the interpretation. Mortality represents a mix of pneumonia incidence and case fatality (11). Conversely, large observational studies have reported a reduction in pneumonia mortality, suggesting a beneficial effect of ICS (12–14). This might be due to reduced pro-inflammatory response during pneumonia with less organ dysfunction and therefore better clinical outcomes (9, 12–14). Alternatively, Suissa suggested that the observed mortality reductions might be explained by collider bias, i.e., hospitalization with milder pneumonia events or uncontrolled beneficial prognostic factors among ICS users (15).

We therefore undertook a large population-based study in Denmark to evaluate ICS use as a prognostic factor for clinical outcomes in COPD patients hospitalized for CAP. To overcome possible collider bias, we controlled meticulously for confounders, and also included a COPD comparison group consisting of former ICS users who likely have a risk factor profile close to that of current ICS users with COPD.

## Materials and methods

### Setting and study cohort

We conducted our cohort study in Northern Denmark, which has a mixed rural/urban population of 1.8 million persons. The cohort consisted of all adult patients with a first-time incident hospital admission with a primary discharge diagnosis of pneumonia, as recorded in regional population-based medical databases between January 1, 1997 and October 25, 2013. In Denmark, the annual incidence of hospitalized CAP, calculated on the basis of the population is 3.1:1000 (16). CAP was defined as a first-time incident hospital admission with a primary diagnosis of pneumonia [*International Classification of Diseases, Tenth Revision* (ICD-10): J10–J18, A37, A481, A709], or with a primary diagnosis of lung abscess, pyothorax, or pleural effusion (ICD-10: J85, J86, J90) coded with a secondary diagnosis of pneumonia. To focus on community-acquired infections, we excluded patients with any recent inpatient hospitalization within 1 month before pneumonia admission (17). The index date was defined as the date of hospital admission for pneumonia.

Information on comorbidities, medications used before admission, complications, and death was obtained from medical databases. Since 1968, each Danish resident has been assigned a unique civil registration number, which is used in all health databases and permits unambiguous record linkage among them (18). The following databases were used: The Civil Registration System, which provides information on vital status; the Danish National Patient Registry (DNPR), which maintains records on all hospitalizations since 1977; and the Prescription Databases of the Central Denmark and the North Denmark Regions, which collect data on all prescriptions filled by ambulatory patients. For additional information on the source databases, see Additional File 1.

### Chronic obstructive pulmonary disease patients hospitalized with pneumonia

Within the pneumonia cohort, patients were defined as having COPD if they were ≥ 40 years old with a diagnosis of COPD recorded before or on the index date [ICD-10: COPD (J44.X)]. COPD was further subcategorized according to (1) known duration of COPD, defined as time since first hospital diagnosis of COPD (≤ 1 year, >1 year); (2) history of frequent COPD exacerbation, defined as ≥2 exacerbations treated with oral steroids or ≥ 1 exacerbation leading to hospital admission during the last year (yes, no); and (3) airflow limitation (GOLD 1–4), based on forced expiratory volume in the first second (FEV1) values recorded in the DNPR during the last 2 years (19). For additional information, see Additional File 1.

### Inhaled corticosteroids use

We defined current ICS users as persons who had filled their most recent ICS prescription within 180 days prior to the index date. Former users had redeemed their most recent prescription ≥181 days before the index date. Non-users were persons with no redeemed ICS prescription before the index date (reference group). To explore robustness of findings, we performed a sensitivity analysis by repeating the current user analyzes for patients who filled an ICS prescription within 90 days before the index date. Using mean defined daily doses (DDD) over a 1-year period before the index date, ICS use was categorized as low dose (≤0.5 DDD), medium dose (0.5 to 1 DDD), or high dose (> 1 DDD) (20) (see Supplementary Table S1; Additional File 1).

### Pneumonia outcomes

We defined the following main outcomes: (1) a composite outcome of any in-hospital pleuropulmonary complication including parapneumonic complicated pleural effusion [ICD-10 codes J86 or J90 or the procedural code for pleural drainage (KGAA10), excluding patients with a pneumothorax (ICD-10 code J93) and/or lung abscess (ICD-10 code J85)], coded as a discharge diagnosis during the incident hospitalization for pneumonia; (2) death within 30 days following the index date. Among patients hospitalized starting in 2005, we also looked at (3) risk of intensive care unit (ICU) admission (see Supplementary Table S2; Additional File 1).

### Statistical analysis

We computed the cumulative incidence of pleuropulmonary complications, ICU admissions during the index hospitalization for pneumonia, and cumulative mortality during 30 days post-admission. Unadjusted and adjusted risk ratios (RR) with 95% confidence intervals (CIs) among current and former ICS users were computed for (1) pleuropulmonary complications, (2) death within 30 days, and (3) ICU admission, using ICS non-users as reference. The RRs were adjusted for a range of confounders, using modified Poisson regression for both in-hospital outcomes and 30-day mortality (21) (see Supplementary Tables S3, S4; Additional File 1). In an additional analysis, we used Cox proportional-hazards instead of Poisson regression for the 30-day mortality outcome. We also analyzed cause-specific respiratory and cardiovascular mortality associated with ICS use, considering death from other causes as a competing risk and therefore restricted to 2002–2011 when data from the Danish Registry of Causes of Death were available. We performed several stratified analyzes of outcome risks according to ICS use. We used SAS software version 9.4 (SAS Institute Inc. Cary, NC). The study was approved by the Danish Data Protection Agency (record number 2016–051-000001).

## Results

The total study cohort included 11,368 COPD patients with a first-time diagnosis of CAP between 1997 and 2013. Among them, 6,073 (53.4%) were current ICS users, 1,733 (15.2%) were former users, and 3,562 (31.3%) were non-users. Compared to non-users or former ICS users, current ICS users were of similar age, had fewer comorbid conditions, and had more use of concurrent medications. Current ICS users had longer COPD duration, more frequent exacerbations, and more prevalent use of systemic steroids and inhaled bronchodilators than non-users. Only 19.9% of our study population were using a dual bronchodilator therapy [long-acting beta agonists (LABA) and long-acting muscarinic antagonists (LAMA)], mainly in the ICS group. In the subgroup with available airflow limitation measurements in the registries (6.9% of cohort members), 72.0% of current ICS users were in the GOLD 3 or 4 spirometric grade compared to 43.1% of non-users and 45.5% of former users. Characteristics of the study cohort are summarized in Table 1. Baseline characteristics by outcome are presented in Table 2.

**Table 1:** Baseline characteristics of ICS users and non-users in the overall study cohort of patients with COPD and pneumonia.

	All patients	ICS non-users	ICS current users	ICS former users
	*n* = 11,368 (100%)	*n* = 3,562 (31.3%)	*n* = 6,073 (53.4%)	*n* = 1,733 (15.2%)
Gender				
Female	5,445 (47.9)	1,560 (43.8)	3,075 (50.6)	810 (46.7)
Male	5,923 (52.1)	2,002 (56.2)	2,998 (49.4)	923 (53.3)
Age (years)				
Median [Q1–Q3]	75.1 [67.6–81.2]	75.9 [68.1–82.3]	74.5 [67.3–80.5]	75.8 [68.2–81.2]
40–59	1,169 (10.3)	357 (10.0)	627 (10.3)	185 (10.7)
60–79	6,813 (59.9)	2,007 (56.4)	3,813 (62.8)	993 (57.3)
80+	3,386 (29.8)	1,198 (33.6)	1,633 (26.9)	555 (32.0)
COPD history				
Duration of COPD (months)*	25.2 [1.1–62.8]	8 [0–36.3]	35.3 [7–74.3]	34.4 [7.4–71.7]
Duration of COPD ≤1 year*	4,286 (37.7)	1,939 (54.4)	1,834 (30.2)	513 (29.6)
Patients with frequent exacerbations#	1,847 (16.2)	227 (6.4)	1,369 (22.5)	251 (14.5)
Airflow limitation				
Gold 1 (% of known GOLD)	35 (4.5)	7 (13.7)	21 (3.3)	7 (6.9)
Gold 2 (% of known GOLD)	226 (28.8)	22 (43.1)	156 (24.7)	48 (47.5)
Gold 3 (% of known GOLD)	329 (42.0)	18 (35.3)	282 (44.6)	29 (28.7)
Gold 4 (% of known GOLD)	194 (24.7)	4 (7.8)	173 (27.4)	17 (16.8)
Gold unknown	10,584 (93.1)	3,511 (98.6)	5,441 (89.6)	1,632 (94.2)
Marital status				
Married	5,344 (47.0)	1,586 (44.5)	2,928 (48.2)	830 (47.9)
Never married	813 (7.1)	307 (8.6)	394 (6.5)	112 (6.5)
Divorced	1,462 (12.9)	431 (12.1)	802 (13.2)	229 (13.2)
Widowed	3,748 (33.0)	1,237 (34.8)	1,949 (32.1)	562 (32.4)
Unknown	1 (0.0)	1 (0.0)	0 (0.0)	0 (0.0)
Alcoholism-related disorders	215 (1.9)	94 (2.6)	86 (1.4)	35 (2.0)
Comorbidity				
Myocardal infarction	1,316 (11.6)	432 (12.1)	647 (10.6)	237 (13.7)
Congestive heart failure	2,038 (17.9)	725 (20.3)	963 (15.9)	350 (20.2)
Cerebrovascular disease	1,640 (14.4)	606 (17.0)	751 (12.4)	283 (16.3)
Diabetes	1,085 (9.5)	348 (9.8)	538 (8.9)	199 (11.5)
Renal disease	484 (4.3)	133 (3.7)	249 (4.1)	102 (5.9)
Liver disease	209 (1.8)	68 (1.9)	104 (1.7)	37 (2.1)
Cancer	1,810 (15.9)	547 (15.4)	959 (15.8)	304 (17.5)
AIDS	1 (0)	0 (0.0)	1 (0.0)	0 (0.0)
Modified CCI score				
Low (0)	4,480 (39.4)	1,336 (37.5)	2,553 (42.0)	591 (34.1)
Medium (1–2)	4,659 (41.0)	1,500 (42.1)	2,434 (40.1)	725 (41.8)
High (3+)	2,229 (19.6)	726 (20.4)	1,086 (17.9)	417 (24.1)
Concurrent medication use‡				
Inhaled bronchodilators	7,784 (68.5)	901 (25.3)	5,629 (92.7)	1,254 (72.4)
LAMA only or LABA only	3,463 (30.4)	368 (10.3)	2,551 (42.0)	544 (31.4)
LAMA & LABA	2,266 (19.9)	42 (1.2)	2,063 (34.0)	161 (9.3)
Systemic steroids†	4,674 (41.1)	652 (18.3)	3,312 (54.5)	710 (40.9)
Immunomodulating agents				
Immunosuppressants	153 (1.3)	51 (1.4)	69 (1.1)	33 (1.9)
Antineoplastic agents	46 (0.4)	12 (0.3)	24 (0.4)	10 (0.6)
Cardiometabolic drugs				
Platelet aggregation inhibitors	3,313 (29.1)	912 (25.6)	1,861 (30.6)	540 (31.2)
Vitamin K antagonists	975 (8.6)	300 (8.4)	506 (8.3)	169 (9.7)
Antiarrhythmics	1,552 (13.6)	459 (12.9)	818 (13.5)	275 (15.9)
Diuretics	6,602 (58.1)	1,763 (49.5)	3,776 (62.2)	1,063 (61.3)
Beta blocking agents	2,076 (18.3)	703 (19.7)	1,014 (16.7)	359 (20.7)
Calcium channel blockers	2,367 (20.8)	613 (17.2)	1,366 (22.5)	388 (22.4)
Agents acting on the renin-angiotensin system	2,946 (25.9)	797 (22.4)	1,667 (27.4)	482 (27.8)
Other antihypertensive drugs	123 (1.1)	34 (1.0)	68 (1.1)	21 (1.2)
Insulin	421 (3.7)	139 (3.9)	203 (3.3)	79 (4.6)
Other glucose-lowering drugs	826 (7.3)	228 (6.4)	463 (7.6)	135 (7.8)
Statins	2,116 (18.6)	522 (14.6)	1,237 (20.4)	357 (20.6)
Proton pump inhibitors	2,955 (26.0)	667 (18.7)	1,770 (29.1)	518 (29.9)
Paracetamol	4,304 (37.9)	1,133 (31.8)	2,397 (39.5)	774 (44.6)
NSAIDs within 60 days	1,259 (11.1)	324 (9.1)	732 (12.0)	203 (11.7)
Antibiotics within 10 days	2,585 (22.7)	719 (20.2)	1,470 (24.2)	396 (22.8)
Macrolides	864 (7.6)	212 (6.0)	521 (8.6)	131 (7,6)
Penicillin V	1,264 (11.1)	419 (11.8)	639 (10.5)	206 (11.9)
Penicillin A	302 (2.7)	84 (2.4)	177 (2.9)	41 (2.4)
Penicillin A + enzyme inhibitor	267 (2.3)	41 (1.1)	183 (3.0)	43 (2.5)
Penicillin M	51 (0.4)	14 (0.4)	33 (0.5)	4 (0.2)
Tetracycline	1 (0.0)	1 (0.0)	0 (0.0)	0 (0.0)
Fluoroquinolone	17 (0.1)	3 (0.1)	13 (0.2)	1 (0.1)

Data are presented as no. (%) except for age and duration of COPD [presented as median (Q1–Q3)]. AIDS, Acquired immune deficiency syndrome; CCI, Charlson Comorbidity Index; COPD, Chronic obstructive pulmonary disease; ICS, Inhaled corticosteroids; LABA, Long-acting beta agonists; LAMA, Long-acting muscarinic antagonists; NSAIDs, Non-steroidal anti-inflammatory drugs. ICS current users: patients who had filled their most recent prescription within 180 days prior to their index date. Former users: patients who had redeemed their most recent prescription ≥ 181 days before their index date. Non-users: patients with no redeemed ICS prescriptions before their index date. *Duration of COPD: time elapsed between first occurrence of hospital-coded COPD and the index date. # Frequent exacerbations: ≥ 2 exacerbations with short-term courses of oral corticosteroids or ≥ 1 exacerbation leading to hospital admission during the previous year. ‡ NSAID use within 60 days before hospital admission. Antibiotic use within 10 days before admission. Other concurrent medications within 365 days prior to admission. †Systemic steroid use refers to at least one prescription for systemic steroids during the year before the index date (chronic or occasional use, indication not available in the registry).

**Table 2:** Cumulative risk of pleuropulmonary complications, 30-day mortality, and ICU admission according to baseline characteristics of the overall study cohort of patients with COPD and pneumonia.

	Patients with a pleuropulmonary complication	Patients deceased at 30 days	Patients admitted to ICU	Patients without any of these complications
	*n* = 315/11,368 (2.8%)	*n* = 1,211/11,368 (10.7%)	*n* = 403/6,165 (6.5%)	*n* = 5,057/6,165 (82.0%)
Gender				
Female	134 (2.5)	522 (9.6)	207 (6.9)	2,488 (83.0)
Male	181 (3.1)	689 (11,6)	196 (6.2)	2,569 (82.1)
Age (years)				
Median [Q1–Q3]	72.9 [64.3–79.7]	79.3 [73.3–84.7]	72.7 [64.6–79.7]	75.3 [67.3–81.8]
40–59	56 (4.8)	47 (4.0)	51 (7.7)	558(86.2)
60–79	186 (2.7)	594 (8.7)	255 (7.4)	2,865 (84.2)
80+	73 (2.2)	570 (16.8)	97 (4.7)	1,634 (78.7)
COPD history				
Duration of COPD (months)	27.7 [0.9–68.3]	29.2 [3.6–68.5]	20.7 [0.0–76.5]	35.0 [2.7–83.8]
Duration of COPD ≤1 year*	119 (2.8)	422 (9.8)	185 (8.8)	1,707 (82.0)
Patients with frequent exacerbations#	39 (2.1)	208 (11.3)	218 (5.3)	933 (82.9)
Airflow limitation				
Gold 1	1 (2.9)	3 (8.6)	3 (8.6)	30 (85.7)
Gold 2	9 (4.0)	13 (5.7)	10 (4.4)	199 (89.2)
Gold 3	8 (2.4)	33 (10.0)	16 (4.9)	277 (85.0)
Gold 4	4 (2.1)	22 (11.3)	17 (8.8)	155 (79.9)
Gold unknown	293 (2.8)	1,140 (10.8)	357 (6.6)	4,396 (82.2)
Marital status				
Married	144 (2.7)	478 (8.9)	193 (6.9)	2,318 (83.7)
Never married	30 (3.7)	86 (10.6)	36 (7.5)	390 (83.0)
Divorced	49 (3.3)	128 (8.7)	58 (6.8)	707 (83.6)
Widowed	92 (2.4)	518 (13.8)	116 (5.7)	1,642 (80.6)
Unknown	0 (0.0)	1 (100.0)	0 (0.0)	0 (0.0)
Alcoholism-related disorders	9 (4.2)	20 (9.3)	22 (15.9)	104 (76.5)
Comorbidity				
Myocardal infarction	32 (2.4)	168 (12.8)	41 (5.1)	657 (81.7)
Congestive heart failure	45 (2.2)	300 (14.7)	68 (6.6)	809 (78.7)
Cerebrovascular disease	48 (2.9)	224 (13.7)	59 (5.7)	821 (79.6)
Diabetes	29 (2.7)	117 (10.8)	51 (7.2)	582 (82.8)
Renal disease	16 (3.3)	67 (13.8)	25 (7.5)	266 (79.9)
Liver disease	10 (4.8)	21 (10.0)	10 (7.2)	114 (83.8)
Cancer	58 (3.2)	279 (15.4)	48 (4.2)	917 (80.6)
AIDS	0 (0.0)	0 (0.0)	0 (0.0)	1 (100.0)
Modified CCI score				
Low (0)	119 (2.7)	361 (8.0)	170 (7.8)	1837 (84.6)
Medium (1–2)	133 (2.8)	493 (10.6)	159 (6.3)	2065 (82.8)
High (3+)	63 (2.8)	357 (16.1)	74 (5.0)	1,155 (79.1)
Concurrent medication use‡				
ICS				
Non-users	115 (3.2)	448 (12.6)	142 (8.9)	1,232 (77.7)
ICS current users	156 (2.6)	554 (9.1)	207 (5.7)	3,051 (84.6)
ICS former users	44 (2.5)	209 (12.1)	54 (5.7)	774 (82.4)
Inhaled bronchodilators	205 (2.6)	772 (9.9)	272 (5.8)	3,886 (84.0)
LAMA only or LABA only	74 (2.1)	315 (9.1)	107 (5.5)	1,667 (85.1)
LAMA & LABA	79 (3.5)	208 (9.2)	122 (6.0)	1,697 (84.1)
Systemic steroids†	109 (2.3)	540 (11.5)	150 (6.0)	2,052 (82.2)
Immunomodulating agents				
Immunosuppressants	4 (2.6)	11 (7.2)	3 (2.3)	115 (90.6)
Antineoplastic agents	0 (0.0)	8 (17.5)	2 (22.2)	6 (66.7)
Cardiometabolic drugs				
Platelet aggregation inhibitors	92 (2.8)	392 (11.8)	152 (6.2)	2,004 (80.2)
Vitamin K antagonists	30 (3.1)	121 (12.4)	40 (5.7)	573 (82.0)
Antiarrhythmics	28 (1.8)	258 (16.6)	46 (6.3)	570 (78.5)
Diuretics	170 (2.6)	826 (12.5)	248 (6.7)	3,001(81.2)
Beta blocking agents	71 (3.4)	219 (10.5)	103 (6.3)	1,329 (82.1)
Calcium channel blockers	73 (3.1)	236 (10.0)	100 (6.7)	1,232 (82.8)
Agents acting on the renin-angiotensin system	90 (3.0)	301 (10.2)	150 (7.2)	1,731 (83.1)
Other antihypertensive drugs	0 (0.0)	15 (12.2)	7 (10.9)	52 (81.3)
Insulin	15 (3.6)	40 (9.5)	25 (9.0)	229 (83.0)
Other glucose-lowering drugs	22 (2.7)	77 (9.3)	43 (7.5)	482 (84.4)
Statins	60 (2.8)	174 (8.2)	124 (6.6)	1,590 (84.8)
Proton pump inhibitors	92 (3.1)	348 (11.8)	93 (4.5)	1,712 (83.9)
Paracetamol	124 (2.9)	584 (13.6)	152 (5.8)	2,099 (80.4)
NSAIDs within 60 days	49 (3.9)	118 (9.4)	55 (8.0)	564 (83.1)
Antibiotics within 10 days	73 (2.8)	264 (10.2)	90 (5.8)	1,293 (83.9)
Macrolides	24 (2.8)	60 (6.9)	32 (6.5)	416 (85.4)
Penicillin V	34 (2.7)	122 (9.6)	37 (5.3)	583 (84.4)
Penicillin A	11 (3.6)	51 (16.9)	8 (4.5)	147 (84.0)
Penicillin A + enzyme inhibitor	5 (1.9)	35 (13.1)	17 (6.4)	217 (81.6)
Penicillin M	0 (0.0)	7 (13.7)	1 (3.1)	28 (87.5)
Tetracycline	0 (0.0)	0 (0.0)	0 (0.0)	1 (100.0)
Fluoroquinolone	0 (0.0)	3 (17.6)	0 (0.0)	0 (0.0)

Pleuropulmonary complications and death could be assessed in all 11,368 COPD patients with a first-time diagnosis of community-acquired pneumonia between 1997 and 2013; ICU risk could be assessed in 6,165 COPD patients between 2005 and 2013 when data on ICU admissions had become available in the Danish National Patient Registry. Data are presented as no. (%) except for age and duration of COPD [presented as median (Q1–Q3)]. AIDS, Acquired immune deficiency syndrome; CCI, Charlson Comorbidity Index; COPD, Chronic obstructive pulmonary disease; ICU, Intensive care unit; ICS, Inhaled corticosteroids; LABA, Long-acting beta agonists; LAMA, Long-acting muscarinic antagonists; NSAIDs, Non-steroidal anti-inflammatory drugs. ICS current users: persons who had filled their most recent prescription within 180 days prior to the index date. Former users: persons who had redeemed their most recent prescription ≥ 181 days before the index date. Non-users: persons with no redeemed ICS prescriptions before the index date. Modified CCI score: computed excluding chronic lung disease. *Duration of COPD: time elapsed between first occurrence of hospital-coded COPD and the index date. # Frequent exacerbations: ≥ 2 exacerbations with short-term courses of oral corticosteroids or ≥ 1 exacerbation leading to hospital admission during the previous year. ‡NSAID use within 60 days before hospital admission. Antibiotic use within 10 days before hospital admission. Other concurrent medications within 365 days prior to admission. †Systemic steroid use refers to at least one prescription for systemic steroids during the year before the index date (chronic or occasional use, indication not available in the registry).

### Pleuropulmonary complications

**Figure 1:**
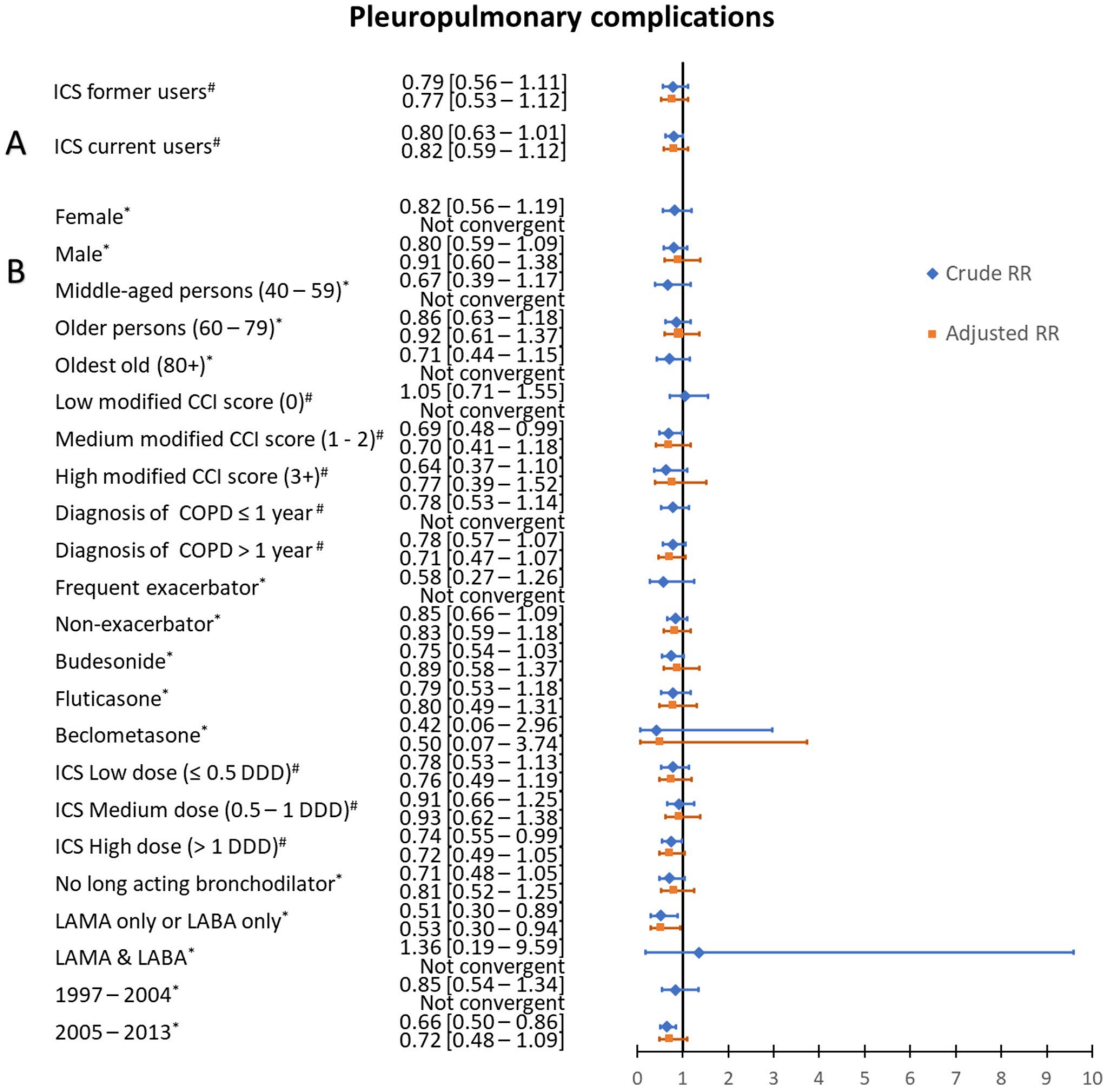
Risk ratios of pleuropulmonary complications of community-acquired pneumonia according to use of inhaled corticosteroids. **(A)** Crude and adjusted risk ratios (RRs) of pleuropulmonary complications of community-acquired pneumonia according to use of inhaled corticosteroids (ICS) among chronic obstructive pulmonary disease (COPD) patients. **(B)** Stratified analysis, in ICS current users, by age category, Charlson Comorbidity Index (CCI) score, duration of COPD, exacerbation history, ICS type, mean defined daily dose, long-acting bronchodilator use, and study period. #Risk ratios for pleuropulmonary complications were adjusted for sex, age, myocardial infarction, congestive heart failure, cerebrovascular disease, diabetes, renal disease, liver disease, cancer, history of alcoholism-related disorders, COPD duration, status of experiencing frequent exacerbations and pre-admission use of antibiotics, non-steroidal anti-inflammatory drugs, paracetamol, systemic steroids, immunosuppressants, inhaled bronchodilators, cardiometabolic drugs, proton pump drugs, and diabetes drugs. *Risk ratios for pleuropulmonary complications were adjusted for sex, age, modified CCI score, history of alcoholism-related disorders, COPD duration, and pre-admission use of antibiotics, non-steroidal anti-inflammatory drugs, paracetamol, systemic steroids or immunosuppressants, inhaled bronchodilators, and cardiometabolic drugs.

Among the 11,368 patients with COPD and CAP, 315 (2.8%) were registered with a pleuropulmonary complication. Proportions of patients with pleuropulmonary complications were 3.2% among ICS non-users, 2.6% among current users, and 2.5% among former users. The crude RR was 0.80 [95% CI: 0.63–1.01] in current ICS users and 0.79 [95% CI: 0.56–1.11] in former users, compared with non-users. The adjusted risk of pleuropulmonary complications was non-significantly decreased with an adjusted risk ratio (aRR) of 0.82 [95% CI: 0.59–1.12] for current ICS users, yet was also decreased at 0.78 [95% CI: 0.55–1.10] for former ICS users (see Figure 1; Supplementary Table S5; Additional File 1).

Stratified analyzes are presented in Figure 1 and Supplementary Table S6, Additional File 1. Compared to the main analysis, we generally observed similar effect sizes with wider CIs. No significant difference was observed according to the ICS type (budesonide, fluticasone and beclomethasone). Using a shorter ICS exposure window of 90 days provided similar results (see Supplementary Table S9; Additional File 1).

### Thirty-day mortality

**Figure 2:**
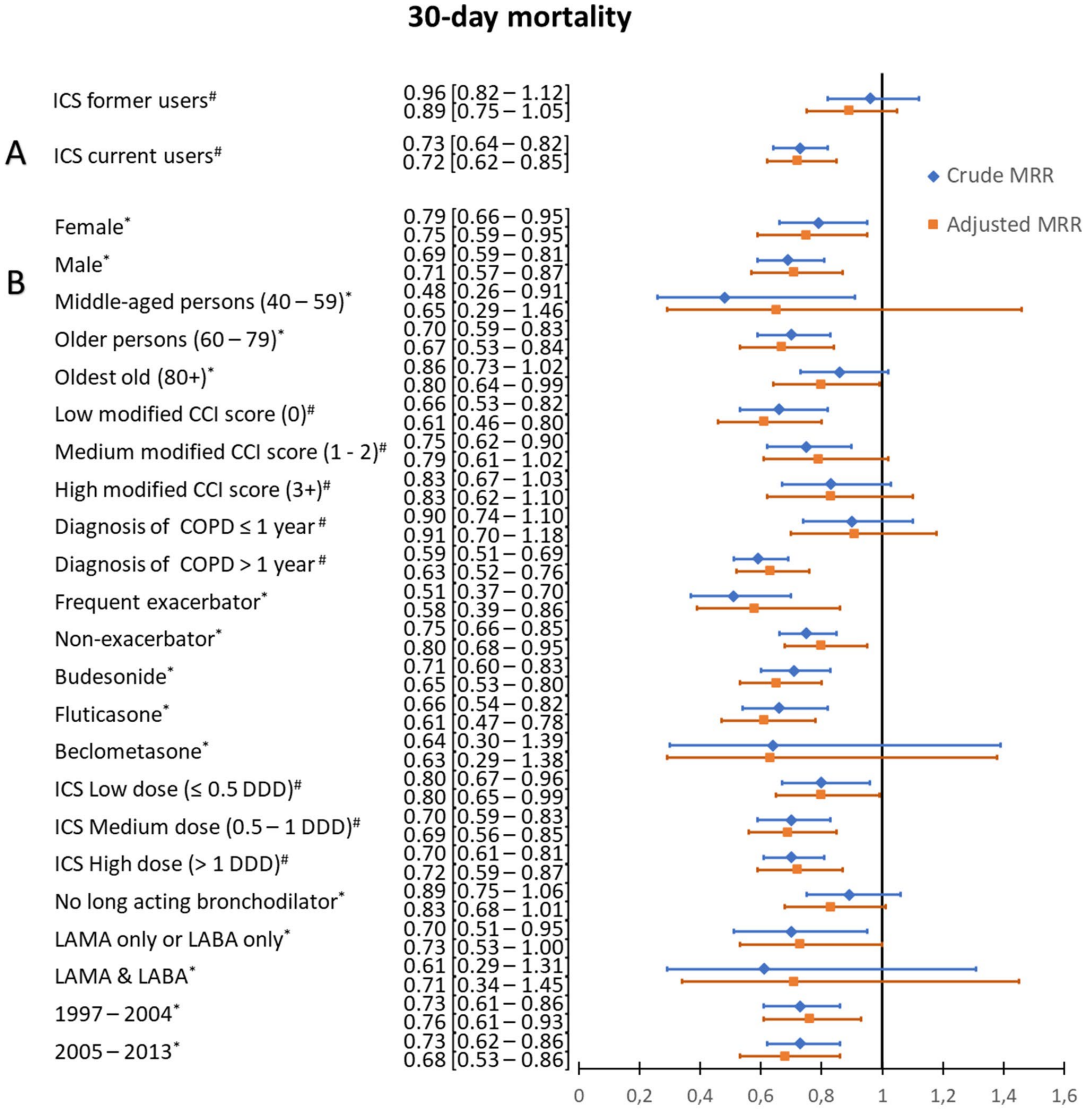
30-day mortality risk ratios following community-acquired pneumonia according to use of inhaled corticosteroids. **(A)** Crude and adjusted 30-day mortality risk ratios (RRs) following community-acquired pneumonia according to use of inhaled corticosteroids (ICS) among chronic obstructive pulmonary disease (COPD) patients. **(B)** Stratified analysis, in ICS current users, by age category, Charlson Comorbidity Index (CCI) score, duration of COPD, exacerbation history, ICS type, mean defined daily dose, long-acting bronchodilator use, and study period. # Risk ratios for 30-day mortality were adjusted for sex, age, myocardial infarction, congestive heart failure, cerebrovascular disease, diabetes, renal disease, liver disease, cancer, history of alcoholism-related disorders, COPD duration, status of experiencing frequent exacerbations, and pre-admission use of antibiotics, non-steroidal anti-inflammatory drugs, paracetamol, systemic steroids, immunosuppressants, inhaled bronchodilators, cardiometabolic drugs, proton pump drugs, and diabetes drugs. *Risk ratios for 30-day mortality were adjusted for sex, age, modified CCI score, history of alcoholism-related disorders, COPD duration, and pre-admission use of antibiotics, non-steroidal anti-inflammatory drugs, paracetamol, systemic steroids or immunosuppressants, inhaled bronchodilators, and cardiometabolic drugs.

Thirty-day mortality was 9.1% in current ICS users, 12.1% in former users, and 12.6% in non-users. Baseline characteristics according to survival at day 30 are presented in Table 3. The crude mortality risk ratio was 0.73 [95% CI: 0.64–0.82] for current ICS users, and 0.96 [95% CI: 0.82–1.12] for former users. The adjusted mortality risk ratio was clearly and significantly decreased for current ICS users [0.72 (95% CI: 0.62–0.85)] and slightly and non-significantly decreased [0.89 (95% CI: 0.75–1.05)] for former users (see Figure 2; Supplementary Table S5; Additional File 1). Stratified analyzes are presented in Figure 2 and Supplementary Table S7, Additional File 1. The lowest aRRs from ICS use were observed in persons with a low modified Charlson Comorbidity Index score [0.61 (95% CI: 0.46–0.80)], duration of COPD >1 year (0.63 [95% CI: 0.52–0.76]), and in persons with frequent exacerbations [0.58 (95% CI: 0.39–0.86)]. The effect sizes were similar across different ICS types and dosages. Among ICS users, stratified analysis showed that the aMRR tended to be lower in patients who used one type of long-acting bronchodilator [LAMA only or LABA only: aMRR = 0.73 (0.53–1.00)] or a dual bronchodilator therapy [LAMA & LABA: 0.71 (0.34–1.45)] compared to those who did not used long-acting bronchodilator [aMRR = 0.83 (0.68–1.01)]. Sensitivity analyzes using a shorter exposure window or a Cox proportional-hazards model yielded similar results (see Supplementary Tables S9, S10; Additional File 1). Analyzes of cause-specific mortality revealed significantly reduced respiratory mortality [adjusted Hazard ratio = 0.71 (0.55–0.92)] and cardiovascular mortality [adjusted Hazard ratio = 0.50 (0.32–0.80)] at 30 days associated with current ICS use (see Supplementary Table S11; Additional File 1).

**Table 3:** Baseline characteristics of ICS users and non-users according to survival at day 30.

	ICS non-users (*n* = 3,562)		ICS users (*n* = 6,073)	
	Alive *n* = 3,114	Dead *n* = 448	Alive *n* = 5,519	Dead *n* = 554
Gender				
Female	1,389 (44.6)	171 (38.2)	2,809 (50.9)	266 (48.0)
Male	1,725 (55.4)	277 (61.8)	2,710 (49.1)	288 (52.0)
Age (years)				
Median [Q1–Q3]	75.3 [67.3–81.7]	79.7 [73.7–85.2]	74.0 [66.8–80.0]	79.1 [73.2–84.3]
40–59	337 (10.8)	20 (4.5)	610 (11.1)	17 (3.1)
60–79	1,796 (57.7)	211 (47.1)	3,531 (64.0)	282 (50.9)
80+	981 (31.5)	217 (48.4)	1,378 (25.0)	255 (46.0)
COPD history				
Duration of COPD (months)	6.9 [0.0–34.3]	16.5 [0.0–47.5]	34.8 [6.9–73.8]	39.7 [7.5–78.1]
Duration of COPD ≤1 year*	1,740 (55.9)	199 (44.4)	1,664 (30.2)	170 (30.7)
Patients with frequent exacerbations#	186 (6.0)	41 (9.2)	1,243 (22.5)	126 (22.7)
Airflow limitation				
Gold 1 (% of known GOLD)	6 (14.0)	1 (12.5)	20 (3.5)	1 (1.9)
Gold 2 (% of known GOLD)	18 (41.9)	4 (50.0)	151 (26.1)	5 (9.3)
Gold 3 (% of known GOLD)	15 (34.9)	3 (37.5)	253 (43.8)	29 (53.7)
Gold 4 (% of known GOLD)	4 (9.3)	0 (0.0)	154 (26.6)	19 (35.2)
Gold unknown	3,071 (98.6)	440 (98.2)	4,941 (89.5)	500 (90.3)
Marital status				
Married	1,408 (45.2)	178 (39.7)	2,719 (49.3)	209 (37.7)
Never married	269 (8.6)	38 (8.5)	351 (6.4)	43 (7.8)
Divorced	384 (12.3)	47 (10.5)	755 (13.7)	47 (8.5)
Widowed	1,053 (33.8)	184 (41.1)	1,694 (30.7)	255 (46.0)
Unknown	0 (0.0)	1 (0.2)	0 (0.0)	0 (0.0)
Alcoholism-related disorders	83 (2.7)	11 (2.5)	79 (1.4)	7 (1.3)
Comorbidity				
Myocardal infarction	375 (12.0)	57 (12.7)	569 (10.3)	78 (14.1)
Congestive heart failure	610 (19.6)	115 (25.7)	827 (15.0)	136 (24.5)
Cerebrovascular disease	514 (16.5)	92 (20.5)	658 (11.9)	93 (16.8)
Diabetes	301 (9.7)	47 (10.5)	490 (8.9)	48 (8.7)
Renal disease	111 (3.6)	22 (4.9)	221 (4.0)	28 (5.1)
Liver disease	62 (2.0)	6 (1.3)	96 (1.7)	8 (1.4)
Cancer	456 (14.6)	91 (20.3)	832 (15.1)	127 (22.9)
AIDS	0 (0.0)	0 (0.0)	1 (0.0)	0 (0.0)
Modified CCI score				
Low (0)	1,198 (38.5)	138 (30.8)	2,379 (43.1)	174 (31.4)
Medium (1–2)	1,314 (42.2)	186 (41.5)	2,208 (40.0)	226 (40.8)
High (3+)	602 (19.3)	124 (27.7)	932 (16.9)	154 (27.8)
Concurrent medication use‡				
Inhaled bronchodilators	783 (25.1)	118 (26.3)	5,123 (92.8)	506 (91.3)
LAMA only or LABA only	325 (10.4)	43 (9.6)	2,343 (42.5)	208 (37.6)
LAMA & LABA	36 (1.2)	6 (1.3)	1,882 (34.1)	181 (32.7)
Systemic steroids†	548 (17.6)	104 (23.2)	2,986 (54.1)	326 (58.8)
Immunomodulating agents				
Immunosuppressants	46 (1.5)	5 (1.1)	64 (1.2)	5 (0.9)
Antineoplastic agents	9 (0.3)	3 (0.7)	21 (0.4)	3 (0.5)
Cardiometabolic drugs				
Platelet aggregation inhibitors	768 (24.7)	144 (32.1)	1,663 (30.1)	198 (35.7)
Vitamin K antagonists	257 (8.3)	43 (9.6)	443 (8.0)	63 (11.4)
Antiarrhythmics	373 (12.0)	86 (19.2)	690 (12.5)	128 (23.1)
Diuretics	1,486 (47.7)	277 (61.8)	3,371 (61.1)	405 (73.1)
Beta blocking agents	618 (19.8)	85 (19.0)	912 (16.5)	102 (18.4)
Calcium channel blockers	533 (17.1)	80 (17.9)	1,250 (22.6)	116 (20.9)
Agents acting on the renin-angiotensin system	696 (22.4)	101 (22.5)	1,509 (27.3)	158 (28.5)
Other antihypertensive drugs	29 (0.9)	5 (1.1)	62 (1.1)	6 (1.1)
Insulin	125 (4.0)	14 (3.1)	185 (3.4)	18 (3.2)
Other glucose-lowering drugs	203 (6.5)	25 (5.6)	423 (7.7)	40 (7.2)
Statins	472 (15.2)	50 (11.2)	1,144 (20.7)	93 (16.8)
Proton pump inhibitors	566 (18.2)	101 (22.5)	1,594 (28.9)	176 (31.8)
Paracetamol	942 (30.3)	191 (42.6)	2,119 (38.4)	278 (50.2)
NSAIDs within 60 days	290 (9.3)	34 (7.6)	670 (12.1)	62 (11.2)
Antibiotics within 10 days	628 (20.2)	91 (20.3)	1,339 (24.3)	131 (23.6)
Macrolides	194 (6.2)	18 (4.0)	489 (8.9)	32 (5.8)
Penicillin V	374 (12.0)	45 (10.0)	584 (10.6)	55 (9.9)
Penicillin A	62 (2.0)	22 (4.9)	156 (2.8)	21 (3.8)
Penicillin A + enzyme inhibitor	33 (1.1)	8 (1.8)	161 (2.9)	22 (4.0)
Penicillin M	11 (0.4)	3 (0.7)	29 (0.5)	4 (0.7)
Tetracycline	1 (0.0)	0 (0.0)	0 (0.0)	0 (0.0)
Fluoroquinolone	2 (0.1)	1 (0.2)	11 (0.2)	2 (0.4)

Data are presented as no. (%) except for age and duration of COPD [presented as median (Q1–Q3)]. AIDS, Acquired immune deficiency syndrome; CCI, Charlson Comorbidity Index; COPD, Chronic obstructive pulmonary disease; ICU, Intensive care unit; ICS, Inhaled corticosteroids; LABA, Long-acting beta agonists; LAMA, Long-acting muscarinic antagonists; NSAIDs, Non-steroidal anti-inflammatory drugs. ICS current users: persons who had filled their most recent prescription within 180 days prior to the index date. Former users: persons who had redeemed their most recent prescription ≥ 181 days before the index date. Non-users: persons with no redeemed ICS prescriptions before the index date. Modified CCI score: computed excluding chronic lung disease. *Duration of COPD: time elapsed between first occurrence of hospital-coded COPD and the index date. # Frequent exacerbations: ≥ 2 exacerbations with short-term courses of oral corticosteroids or ≥ 1 exacerbation leading to hospital admission during the previous year. ‡NSAID use within 60 days before hospital admission. Antibiotic use within 10 days before hospital admission. Other concurrent medications within 365 days prior to admission. †Systemic steroid use refers to at least one prescription for systemic steroids during the year before the index date (chronic or occasional use, indication not available in the registry).

### Intensive care unit admission

**Figure 3:**
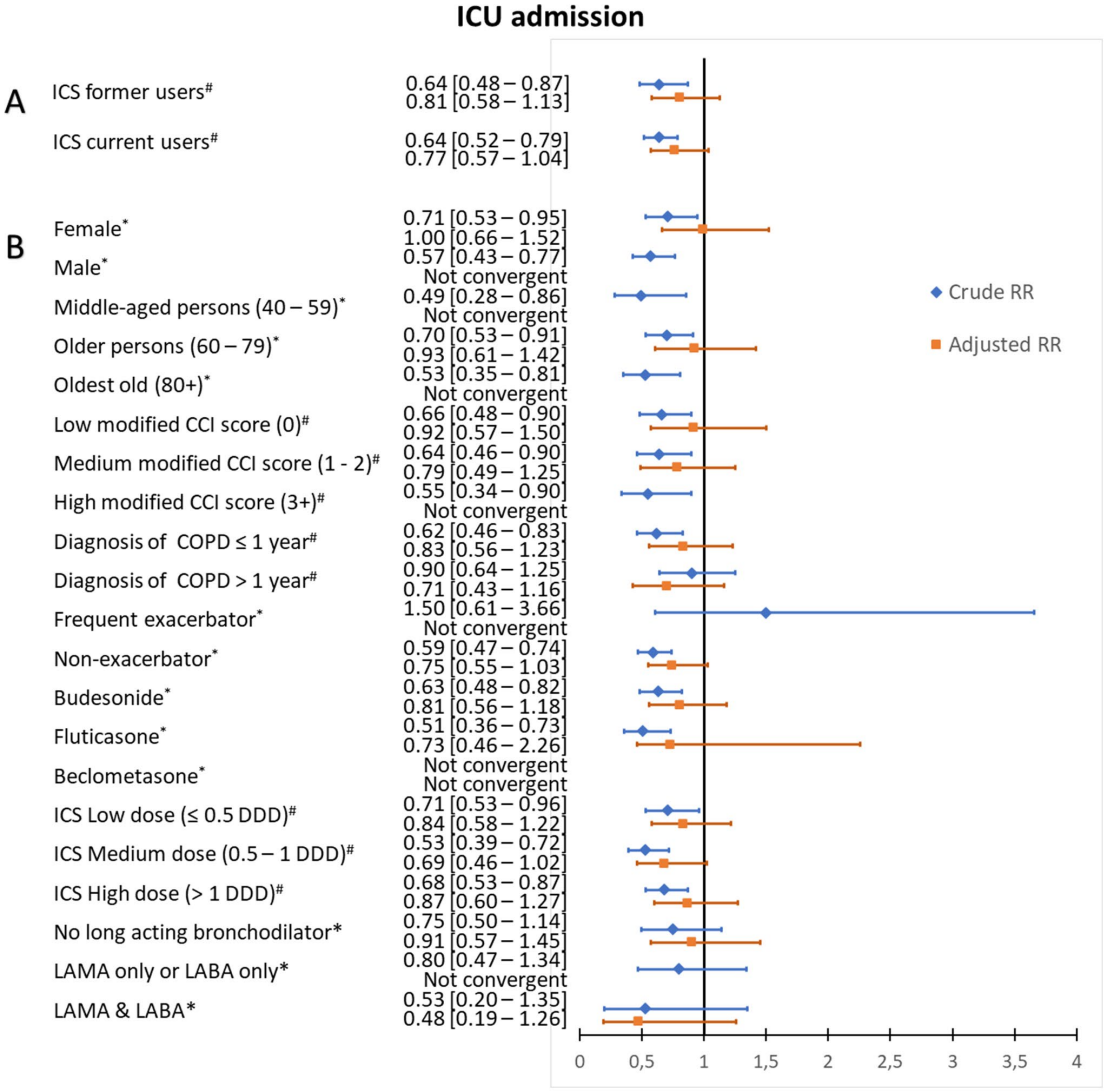
Risk ratios for intensive care unit admission according to use of inhaled corticosteroids. **(A)** Crude and adjusted risk ratios (RRs) for intensive care unit admission of patients with chronic obstructive pulmonary disease (COPD) and community-acquired pneumonia according to use of inhaled corticosteroids (ICS). **(B)** Stratified analysis, among current ICS users, by age category, Charlson Comorbidity Index (CCI) score, duration of COPD, exacerbation history, ICS type, mean defined daily dose, and long-acting bronchodilator use. # Risk ratios for intensive care unit admission were adjusted for sex, age, myocardial infarction, congestive heart failure, cerebrovascular disease, diabetes, renal disease, liver disease, cancer, history of alcoholism-related disorders, COPD duration, status as a patient with frequent exacerbations, and pre-admission use of antibiotics, non-steroidal anti-inflammatory drugs, paracetamol, systemic steroids, immunosuppressants, inhaled bronchodilators, cardiometabolic drugs, proton pump drugs, and diabetes drugs. *Risk ratios for intensive care unit admission were adjusted for sex, age, modified CCI score, history of alcoholism-related disorders, COPD duration, and pre-admission use of antibiotics, non-steroidal anti-inflammatory drugs, paracetamol, systemic steroids or immunosuppressants, inhaled bronchodilators, and cardiometabolic drugs.

Among the 6,165 COPD patients who had available data on ICU admissions in the DNPR during 2005–2013, 403 (6.5%) were admitted to an ICU (Table 2). The ICU admission rate was 5.7% both among current ICS users and former users, compared to 8.9% among non-users. The crude risk ratio for ICU admission was significantly decreased for current users [0.64 (95% CI: 0.52–0.79)] but also for former users [0.64 (95% CI: 0.48–0.87)]. After adjustment for confounders, the aRRs for ICU admission were non-significantly decreased for current ICS users [0.77 (95% CI: 0.57–1.04)], and for former users [0.81 (95% CI: 0.58–1.13)] (see Figure 3; Supplementary Table S5; Additional File 1). Stratified and additional analyzes are presented in Figure 3 and Supplementary Tables S8, S9, Additional File 1. No significant difference was observed according to the ICS type (budesonide, fluticasone).

## Discussion

We found an approximately 20% reduced risk of pleuropulmonary complications and ICU admission in COPD patients with CAP who were current ICS users. However, these reductions were statistically non-significant and were also observed among former ICS users. Conversely, we found a stronger and significant association between current ICS use and decreased 30-day mortality from CAP, especially among patients with frequent exacerbations, which was not observed in former ICS users.

Our results for a numerically reduced risk of pleuropulmonary complications are consistent with those reported by Sellares et al., who observed a lower incidence of complicated parapneumonic effusion in current ICS users [1.4% versus 4.2%; odds ratio = 0.32 (0.12–0.90)] (4). However, we observed a similarly lowered risk of complications among former ICS users, suggesting that this association may not be related exclusively to acute ICS effects but also to possible unmeasured confounders, such as pneumonia severity or patient frailty. Compared to ICS non-users, both current and former ICS users had longer COPD duration and more frequent exacerbations, which should not be prognostically beneficial *per se*. However, longer time since diagnosis of COPD may be related to survivor bias. Also, uncontrolled confounding by contraindication for ICS use, for example polypharmacy and general frailty (i.e., short life expectancy), may have played a role. Compared to ICS non-users, current and former ICS users also may have had a higher baseline risk of pneumonia, and been admitted with less severe pneumonia less likely leading to complications (surveillance bias) (22). This is consistent with the finding that both current and former ICS users were less likely to be admitted to the ICU (5–10).

Compared to ICS non-users, we observed a significantly decreased 30-day mortality among current ICS users [aRR 0.72 (95% CI: 0.62–0.85)], while mortality was not materially decreased among former ICS users. This difference in mortality was consistent during the periods 1997–2004 and 2005–2013. In comparison, a meta-analysis of six RCTs by Festic et al. reported a slightly decreased, yet imprecise, risk ratio of 0.91 [0.52–1.59] for pneumonia fatality (pneumonia deaths/number of COPD patients with pneumonia) (9). More recently, in an analysis of 16 RCTs, Almagro et al. reported an RR of 0.97 [0.58–1.60] for pneumonia-related mortality, thus mixing pneumonia incidence and fatality (pneumonia deaths/total number of COPD patients) (11). In addition to statistical imprecision, the results of the RCTs were limited by short follow-up (1 year in most cases) and highly selected populations that may differ significantly from COPD patients seen in routine clinical practice (11, 23). These limitations also may explain the much lower pneumonia fatality rate of 3% (pooled results of the 16 RCTs) compared to 10.7% in our population-based study. In contrast, three previous large observational studies reported a reduction in pneumonia fatality associated with ICS use (odds ratio for 30-day mortality varying from 0.74 to 0.80), consistent with our findings (12–14). These three studies included 36,116 COPD patients identified from U.S. Veterans Affairs databases. Despite their large sample sizes, these studies had some limitations: (1) adjustment for previous exacerbations was reported only in the study by Joo et al. (12), (2) none of the studies could account for ICS type or dosage, and (3) more than 98% of the included patients were male, compared to 52.1% males in our COPD population. This high proportion of women in our study is representative of the sex ratio observed in Danish patients with COPD (24). The prevalence of COPD in Denmark is among the highest in the Western world. It has been estimated that ~400,000 of the total population of ~5.5 million have obstructive lung function impairment compatible with COPD (25). ICS use among COPD patients in Denmark is known to be as high as 70% (26).

Since ICS are infrequently used as monotherapy in COPD, and are most often used in combination with LABA or LAMA, any association of ICS with mortality might be confounded by a potential beneficial impact of these bronchodilators. We therefore adjusted for bronchodilator use (presence or absence) in our analysis, and still observed a clearly lower mortality with ICS use. Among ICS users, stratified analysis showed that the aMRR tended to be lower in patients who used one type of long-acting bronchodilator [LAMA only or LABA only: aMRR = 0.73 (0.53–1.00)] or a dual bronchodilator therapy [LAMA & LABA: 0.71 (0.34–1.45)] compared to those who did not used long-acting bronchodilator [aMRR = 0.83 (0.68–1.01)]. Of note, only 19.9% of our study population were receiving a dual bronchodilator therapy (LAMA & LABA). This low prevalence is partly explained by our study period (1997–2013), that started way before the first LAMA therapy was approved in Europe (Tiotropium HandiHaler® in 2002).

One of our major findings was that among COPD patients without frequent exacerbations, ICS users had only a modest (although significant) decrease in pneumonia mortality [aRR 0.80 (95% CI: 0.68–0.95)], whereas frequent exacerbators with ICS use had an aRR as low as 0.58 [95% CI: 0.39–0.86]. Although other potential risks and benefits of ICS must obviously be considered in any treatment decision, our results are in line with current COPD treatment guidelines indicating that ICS should be used in patients with a history of exacerbations despite optimal treatment with long-acting bronchodilators (19, 27).

Another interesting finding is that 30-day mortality following pneumonia was only slightly affected by ICS type or dosage and even tended to be lowest with higher ICS doses, while previous studies have shown that these parameters affect the risk of pneumonia (5, 28). To the best of our knowledge, our study is the first to highlight this point. While our study cannot provide a detailed biological explanation of the mechanisms by which ICS may reduce pneumonia mortality among COPD patients, a recently published RCT on the role of systemic hydrocortisone among patients hospitalized for severe community-acquired pneumonia has indicated that those treated with hydrocortisone had a lower risk of death by day 28 than those in the placebo group, suggesting a protective effect through an antiinflammatory and immunomodulatory effects of glucocorticoids (29). Similar to systemic steroids, previous studies have shown that ICS treatment decreases the systemic levels of a number of inflammatory biomarkers in COPD patients like CRP level, serum levels of soluble TNF receptor-2. ICS also decrease the local response with lower sputum differential neutrophils count and total eosinophil count, bronchial CD45+ and CD4+ cells, and cells expressing genes for tumor necrosis factor-α and IFN-γ (30). By reducing the local and systemic pro-inflammatory response during pneumonia, ICS may lead to less hyperinflammation and organ dysfunction and, therefore, better clinical outcomes (30–33). As for an example, the presence of microorganisms during pneumonia leads to increased leukocyte migration that may cause a variety of lung diseases and injuries (13). This effect may be counterbalanced by ICS treatment, preventing excess sequestration and subsequent lung injury. This hypothesis is in line with our observation that mortality tended to be lowest with higher ICS doses suggesting more decreased inflammatory response. Whereas the basic mechanism of action is similar for all ICS, the pharmacokinetic and characteristics of ICS are quite different in terms of receptor affinity, bioavailability, lipophilicity and drug persistence in the airways (34). Interestingly, we did not observed variation across the different ICS type while the immunosuppressant potency of fluticasone is reported to be up to 10-fold higher than that of budesonide with regard to *ex vivo* inhibition of human alveolar macrophage innate immune response to bacterial triggers (35).

The positive predictive value (PPV) of ICU admission codes in the DNPR is 87.2% (95% CI: 75.6–94.5) (36). The fact that ICU admission risk tended to be reduced both among current and former ICS users may suggest unmeasured confounding. In clinical practice, ICU admission is offered to patients who are expected to have a clear prognostic benefit from invasive monitoring and treatment (37). In addition, the patient’s quality of life, functional level at home, and hospital capacity may influence the decision to admit a patient to an ICU. Thus, use of ICU admission as an outcome is challenging in observational prognostic studies.

Strengths of our study include its large sample size, use of routinely collected clinical data, and a population-based design with access to individual-level information. Follow-up was virtually complete for pleuropulmonary outcomes and death. The DNPR maintains records on all hospitalizations since 1977, including dates of admission and discharge, and up to 20 discharge diagnoses, coded by physicians according to ICD-10. A previous study, based on medical history, clinical symptoms and findings, and spirometry results reported that the PPV of acute COPD discharge diagnoses is 92% (95% CI: 91–93%) for presence of underlying COPD (38). Although not based on medical charts review, the validity of diagnosis of pneumonia in our study is also expected to be high. A previous study based on clinical, laboratory and radiological findings indeed reported a positive predictive value of 90% (95% CI: 82–95%) for diagnosis of pneumonia in Danish medical databases (39).

Our study also has limitations. Our observation that ICS users had fewer comorbidities but more concurrent medications may suggest better treatment, better access to medical care in general, and perhaps higher socioeconomic and educational status. We lacked data on socioeconomic variables, on markers of pneumonia severity (severity scores and blood gas results) and on hospital medications that could have had an impact on the outcomes, e.g., systemic steroid use and antibiotics. All of these variables could represent potential confounders that we were unable to adjust for. We had no data on blood eosinophil count, which may predict the magnitude of any ICS effect in preventing future exacerbations; of note, the GOLD guidelines proposed a threshold of >300 Eosinophilic cells/μl for initiating ICS treatment in combination with LAMA or LABA (40). Assessment of blood eosinophils as a covariate in observational studies that use historical routine clinical care data is challenging, since few COPD patients have eosinophils measured or have persistently high eosinophils over time (41, 42). Lastly, as this large epidemiological study was based on medical and administrative databases, without access to patient’s medical records, we were also unable to confirm that the redeemed ICS prescriptions were actually used. Nevertheless, because the prescription data are prospectively recorded, any misclassification of ICS use due to nonadherence would generally bias measures of associations toward the null.

Unmeasured confounding by indication for ICS use may be present in our study since our adjusted analysis could not fully account for reduced FEV1, as information on compromised airflow was missing for many patients (these data were only voluntarily reported in medical databases we based on). However, it is unlikely that lung function would be associated with both pneumonia outcomes and use of ICS independently of the many other markers of COPD severity for which we were able to adjust. Still, as in any observational study, residual confounding may have affected our risk estimates.

## Conclusion

Among COPD patients with CAP, we observed a modest and non-significantly decreased risk of pleuropulmonary complications and ICU admission among current ICS users. This was also seen in former ICS users however, and may be driven by residual confounding. In contrast, 30-day mortality was significantly decreased among current ICS users but not former users, particularly among those with frequent exacerbations. Our results are consistent with current GOLD recommendations suggesting that ICS use be limited to patients with a history of exacerbations despite optimal treatment with long-acting bronchodilators.

## Data availability statement

The datasets presented in this article are not readily available because Danish data protection legislation does not allow researchers to share raw data from the registries with third parties. The data sources used in this study can be accessed by researchers through application to the Danish Data Protection Agency and the Danish Health Data Authority. Requests to access the datasets should be directed to Danish Data Protection Agency and the Danish Health Data Authority.

## Ethics statement

The studies involving human participants were reviewed and approved by Danish Data Protection Agency (record number 2016–051-000001). Written informed consent for participation was not required for this study in accordance with the national legislation and the institutional requirements.

## Author contributions

DB, LW, RT, JM, NS, PD, CA, VJ, and HS contributed substantially to the study design, data analysis and interpretation, and the writing of the manuscript. All of the authors critically revised the manuscript for important intellectual content and gave final approval for the version to be published and agree to be accountable for all aspects of the work in ensuring that questions related to the accuracy or integrity of any part of the work are appropriately investigated and resolved.

## Funding

This work was supported by a research grant from the Fonds de Recherche en Santé Respiratoire and the Fondation du Souffle [FR2017]. The funding body had no role in the design of the study, collection, analysis, interpretation of data, nor in writing the manuscript.

## Conflict of interest

The authors declare that the research was conducted in the absence of any commercial or financial relationships that could be construed as a potential conflict of interest.

## Publisher’s note

All claims expressed in this article are solely those of the authors and do not necessarily represent those of their affiliated organizations, or those of the publisher, the editors and the reviewers. Any product that may be evaluated in this article, or claim that may be made by its manufacturer, is not guaranteed or endorsed by the publisher.

## Abbreviations

95% CI: 95% confidence interval
aRR: Adjusted risk ratio
CAP: Community-acquired pneumonia
CI: Confidence interval
COPD: Chronic obstructive pulmonary disease
DDD: Defined daily dose
DNPR: Danish National Patient Registry
FEV1: Forced expiratory volume in the first second
ICD-10: *International Classification of Diseases, Tenth Revision*
ICS: Inhaled corticosteroids
ICU: Intensive care unit
LABA: Long-acting beta agonists
LAMA: Long-acting muscarinic antagonists
PPV: Positive predictive value
RCT: Randomized controlled trial
RR: Risk ratio
