# Supplementary material for: Inhaled corticosteroids and adverse outcomes among chronic obstructive pulmonary disease patients with community-acquired pneumonia: a population-based cohort study
Source: Front Med (Lausanne). 2023 Jul 24;10:1184888. doi: 10.3389/fmed.2023.1184888 (PMC10405521; doi:10.3389/fmed.2023.1184888)
Supplement: Supplementary file 1 [file Table_1.DOCX]

***Supplementary Material***

**Inhaled corticosteroids and adverse outcomes among chronic obstructive pulmonary disease patients with community-acquired pneumonia: a population-based cohort study**

**Damien Basille*, Lei Wang, Reimar Wernich Thomsen, Jyothi Menon, Nisha Shetty, Pierre Duhaut, Claire Andrejak, Vincent Jounieaux, Henrik Toft Sørensen**

*** Correspondence:** Damien Basille: basille.damien@chu-amiens.fr

**Index**

[Additional data on Patients and methods 3](#_Toc138201418)

[Information contained in Danish databases 3](#_Toc138201419)

[Exacerbation definition 4](#_Toc138201420)

[Confounding factors 5](#_Toc138201421)

[References 6](#_Toc138201422)

[Supplementary tables 7](#_Toc138201423)

[Table S1. ATC codes for ICS and defined daily doses (based on the World Health Organization classification). 7](#_Toc138201424)

[Table S2. Definition of the outcomes. 8](#_Toc138201425)

[Table S3. Modified Charlson comorbidity index weights and codes (chronic pulmonary diseases are excluded). 9](#_Toc138201426)

[Table S4. Anatomical Therapeutic Chemical (ATC) codes. 10](#_Toc138201427)

[Table S5. Pleuropulmonary complication, 30-day mortality, and intensive care unit admission rates after CAP admission, according to ICS use. 12](#_Toc138201428)

[Table S6. Stratified analysis of the pleuropulmonary complication rate according to ICS use, by sex, age, CCI score, COPD duration, exacerbation history, ICS types, mean DDD, long-acting bronchodilator use, and study period. 13](#_Toc138201429)

[Table S7. Stratified analysis of the 30-day mortality risk ratio according to ICS use, by sex, age, CCI score, COPD duration, exacerbation history, ICS types, mean DDD, long-acting bronchodilator use, and study period. 16](#_Toc138201430)

[Table S8. Stratified analysis of the ICU admission rate according to ICS use, by sex, age, CCI score, COPD duration, exacerbation history, ICS types, mean DDD, and long-acting bronchodilator use. 19](#_Toc138201431)

[Table S9. Pleuropulmonary complication, 30-day mortality, and intensive care unit admission rates after CAP admission, according to ICS use. Sensitivity analysis using a 90 days exposure window. 22](#_Toc138201432)

[Table S10. 30-day mortality rate after CAP admission, according to ICS use. Sensitivity analysis using a Cox proportional-hazards model instead of Poisson model. 23](#_Toc138201433)

[Table S11. Risk of all-cause mortality, respiratory mortality and cardiovascular mortality among a subcohort of 6,917 COPD patients followed from Jan 1, 2002, to Dec 31, 2011. 24](#_Toc138201434)

# Additional data on **Patients and methods**

## Information contained in Danish databases

The Danish National Health Service provides universal, tax-supported health care, including free access to primary and hospital care and reimbursement of most prescription medication costs. Since 1968, each Danish resident has been assigned a unique civil registration number, which encodes sex and birth date, which is used in all health databases and permits unambiguous record linkage among them [1].

The Civil Registration System (CRS) is an administrative register. It contains individual-level information on all persons residing in Denmark. It encompasses persons who have lived in Denmark since 1968. By January 2014, the CRS had cumulatively registered 9.5 million individuals with more than 400 million person-years of follow-up. A unique ten-digit Civil Personal Register number assigned to all persons in the CRS allows for technically easy, cost-effective, and unambiguous individual-level record linkage among Danish registers. Daily updated information on migration and vital status allows for nationwide cohort studies with virtually complete long-term follow-up for emigration and death [2].

The Danish National Patient Registry (DNPR) maintains records on all hospitalizations since 1977, including dates of admission and discharge, and up to 20 discharge diagnoses, coded by physicians according to the *International Classification of Diseases, Tenth Revision* (ICD-10) during the study period [3]. The DNPR provides a data source for identifying diseases, examinations, certain in-hospital medical treatments, and surgical procedures. DNPR data are linkable at the patient level with data from other Danish administrative registries, and clinical registries via Civil Personal Register numbers.

The Prescription Databases of the Central Denmark and the North Denmark Regions (PDCDNDR) collect data on all prescriptions filled by ambulatory patients. The PDCDNDR forwards data on reimbursable medicine to their local regional Health Service sections on a monthly basis [4]. The main variables are the Civil Personal Register numbers of the patient, name of the drug, ATC code, package identifier (enabling identification of brand, quantity, and formulation of the drug), date of refill, code identifying the prescribing physician, and code identifying the dispensing pharmacy. The PDCDNDR is linked to other Danish registries via Civil Personal Register numbers.

## Exacerbation definition

chronic obstructive pulmonary disease (COPD) exacerbation was defined based on short-term courses of oral corticosteroids or acute COPD admissions within one year before index date.

Short-term course of oral corticosteroids:

A COPD exacerbation was designated when a patient filled a prescription for a short-term course of oral corticosteroids (a maximum of 20 tablets of 25 mg in one claim of either prednisolone (ATC-code H02AB06) or prednisone (H02AB07)) during the year before the index date. To be considered a new COPD exacerbation, two prescriptions had to be ≥ 28 days apart [5,6]. Oral corticosteroid use for reasons other than COPD exacerbation could not be excluded with certainty. However, on a population level, the most frequent indication for short-term oral corticosteroid treatment is respiratory disease [7]. All participants in our study had known hospital-diagnosed COPD, making exacerbation as indication for oral corticosteroids most likely.

Acute COPD admission:

Acute COPD admissions within the previous year were ascertained by searching the DNPR for admissions lasting ≥1 day with a primary discharge diagnosis of COPD (ICD-10 code J44), or primary discharge diagnosis of respiratory failure (J96) or J13-18 (pneumonia) with COPD (J44) as secondary diagnosis [6].

## Confounding factors

We obtained data on comorbidities and other covariates that may be associated with both ICS use and community-acquired pneumonia outcomes from the DNPR and PDCNDR. Using the complete medical history of discharge diagnoses available since 1977, we computed a modified Charlson Comorbidity Index (CCI) (excluding chronic lung disease) score for each patient [8]. The codes and the weights that were used are provided in e-Table 2. Three comorbidity levels were defined: low (score of 0), medium (score of 1–2), and high (score ≥3). We also ascertained conditions not included in the CCI: history of alcoholism-related disorders (ICD-10 codes F10, K86.0, Z72.1, R78.0, T51, K29.2, G62.1, G72.1, and G31.2), use of immunosuppressants, glucocorticoids, or paracetamol within the year before the admission for pneumonia, use of non-steroidal anti-inflammatory drugs within 60 days before the admission, use of systemic antibiotics within 10 days before the admission, and use of other drugs related to relevant major comorbidities (*i.e*., chronic respiratory disorder, cardiovascular disease, and diabetes). Regarding inhaled therapeutics, we adjusted analysis on use of inhaled bronchodilators (presence or absence). To take into account the exact nature of the inhalatory therapy, we also performed stratified analysis on the type of bronchodilators that were used (No long-acting bronchodilators, long-acting muscarinic antagonists (LAMA) only or long-acting muscarinic antagonists (LABA) only, LAMA & LABA). The Anatomical Therapeutic Chemical (ATC) Classification System codes for each medication are provided in e-Table 3.

# References

1. Schmidt M, Schmidt SAJ, Adelborg K, Sundbøll J, Laugesen K, Ehrenstein V, et al. The Danish health care system and epidemiological research: from health care contacts to database records. Clin. Epidemiol. 2019;Volume 11:563–91.

2. Schmidt M, Pedersen L, Sørensen HT. The Danish Civil Registration System as a tool in epidemiology. Eur. J. Epidemiol. 2014;29:541–9.

3. Schmidt M, Schmidt SAJ, Sandegaard JL, Ehrenstein V, Pedersen L, Sørensen HT. The Danish National Patient Registry: a review of content, data quality, and research potential. Clin. Epidemiol. 2015;7:449–90.

4. Ehrenstein V, Antonsen S, Pedersen L. Existing data sources for clinical epidemiology: Aarhus University Prescription Database. Clin. Epidemiol. 2010;2:273–9.

5. Lange P, Tøttenborg SS, Sorknæs AD, Andersen JS, Søgaard M, Nielsen H, et al. Danish Register of chronic obstructive pulmonary disease. Clin. Epidemiol. 2016;8:673–8.

6. Ingebrigtsen TS, Marott JL, Lange P, Hallas J, Nordestgaard BG, Vestbo J. Medically treated exacerbations in COPD by GOLD 1-4: A valid, robust, and seemingly low-biased definition. Respir. Med. 2015;109:1562–8.

7. van Staa TP. Use of oral corticosteroids in the United Kingdom. QJM 2000;93:105–11.

8. Charlson ME, Pompei P, Ales KL, MacKenzie CR. A new method of classifying prognostic comorbidity in longitudinal studies: development and validation. J. Chronic Dis. 1987;40:373–83.

# Supplementary tables

## Table S1. ATC codes for ICS and defined daily doses (based on the World Health Organization classification).

| Medication | ATC codes | | Defined daily dose  (DDD) | |
| --- | --- | --- | --- | --- |
|  | ICS | Combination (ICS + LABA) | Administration | DDD |
| Beclometasone | R03BA01 | R03AK08 R03AK13 | Powder / spray  Inhaled solution | 0,8 mg  1,5 mg |
| Budesonide | R03BA02 | R03AK07 R03AK12 | Powder / spray  Inhaled solution | 0,8 mg  1,5 mg |
| Fluticasone / Fluticasone furoate | R03BA05  R03BA09 | R03AK06  R03AK10  R03AK11 | Powder / spray  Inhaled solution | 0,6 mg  1,5 mg |
| Mometasone | R03BA06 | R03AK09 | Powder | 0,4 mg |

Abbreviations: ATC = Anatomical therapeutic chemical; ICS = Inhaled corticosteroids; LABA = Long-acting β2-agonists; DDD = Defined daily dose

## Table S2. Definition of the outcomes.

| **Outcomes** | **Source** | **Inclusion codes** | **Exclusion codes** |
| --- | --- | --- | --- |
| **Pleuropulmonary complications** |  |  |  |
| Parapneumonic complicated pleural effusion | DNPR/ICD-10  DNPR/Procedure | J86, J90  KGAA10 | J93 |
| Lung abscess | DNPR/ICD-10 | J85 |  |
| **Intensive care unit admission** | DNPR/Procedure | NABE, NABB |  |
| **Death** | Danish Civil  Registration System |  |  |

## Table S3. Modified Charlson comorbidity index weights and codes (chronic pulmonary diseases are excluded).

| **Disease** | **Weight** | **ICD-10 codes** |
| --- | --- | --- |
| Myocardial infarction | 1 | I21, I22, I23 |
| Congestive heart failure | 1 | I50, I11.0, I13.0, I13.2 |
| Peripheral vascular disease | 1 | I70-I74, I77 |
| Cerebrovascular disease | 1 | I60-I69, G45, G46 |
| Dementia | 1 | F00-F03, F05.1, G30 |
| Connective tissue disease | 1 | M05, M06, M08, M09, M30-M36, D86 |
| Ulcer disease | 1 | K22.1, K25-K28 |
| Mild liver disease | 1 | B18, K70.0-K70.3, K70.9, K71, K73, K74, K76.0 |
| Diabetes types I and II | 1 | E10.0, E10.1, E10.9, E11.0, E11.1, E11.9 |
| Hemiplegia | 2 | G81, G82 |
| Moderate to severe kidney disease | 2 | I12, I13, N00-N05, N07, N11, N14, N17-N19, Q61 |
| Diabetes with end-organ damage | 2 | E10.2-E10.8, E11.2-E11.8 |
| Any tumor | 2 | C00-C75 |
| Leukemia | 2 | C91-C95 |
| Lymphoma | 2 | C81-C85, C88, C90, C96 |
| Moderate to severe liver disease | 3 | B15.0, B16.0, B16.2, B19.0, K70.4, K72, K76.6, I85 |
| Metastatic solid tumor | 6 | C76-C80 |
| Acquired immune deficiency syndrome | 6 | B21-B24 |

## Table S4. Anatomical Therapeutic Chemical (ATC) codes.

|  | Non-steroidal anti-inflammatory drugs | M01A |
| --- | --- | --- |
|  | Acetylsalicylic acid high dose | N02BA01 |
|  | Glucocorticoids for systemic use | H02AB, H02B |
| Inhaled bronchodilators | Adrenergics, inhalants | R03AC |
|  | Adrenergics + corticosteroids, inhalants | R03AK06 to R03AK13 |
|  | Adrenergics + anticholinergics | R03AL |
|  | Anticholinergics | R03BB |
|  | Immunosuppressants | L04 |
|  | Antineoplastic agents | L01 |
| Antibiotics | Macrolides | J01FA |
|  | Penicillin V | J01CE02 |
|  | Penicillin A | J01CA01, J01CA04 |
|  | Penicillin A + beta-lactamase inhibitors | J01CR01, J01CR02 |
|  | Penicillin M | J01CF |
|  | Tetracyclines | J01A |
|  | Fluoroquinolones | J01MA |
|  | First-generation cephalosporins | J01DB |
|  | Second-generation cephalosporins | J01DC |
|  | Third-generation cephalosporins | J01DD |
|  | Paracetamol | N02BE01 |
| Cardiovascular drugs | Platelet aggregation inhibitors | B01AC |
|  | Vitamine K antagonists | B01AA |
|  | Antiarrhythmics | C01A, C01B |
|  | Diuretics | C03, C07B, C07D, C09DA, C09XA52, C09XA54, C08G, C09DX01, C09DX03 |
|  | Beta blocking agents | C07 |
|  | Calcium channel blockers | C08, C09BB, C09DB, C09DX01, C09DX03, C09XA53 |
|  | Agents acting on the renin-angiotensin system | C09 |
|  | Other Antihypertensives | C02 |
|  | Lipid modifying agents | C10 |
|  | Insulin | A10A |
|  | Blood glucose lowering drugs, excl. insulins | A10B |
|  | Proton pump inhibitors | A02BC |

## Table S5. Pleuropulmonary complication, 30-day mortality, and intensive care unit admission rates after CAP admission, according to ICS use.

| **Pleuropulmonary complications** | | | | | |
| --- | --- | --- | --- | --- | --- |
|  | **All patients** | **No. of Events** | **%** | **Crude RR**  **(95% CI)** | **Adjusted RR**  **(95% CI)** |
| ICS non-users | 3,562 | 115 | 3.2 | 1 (referent) | 1 (referent) |
| ICS current users | 6,073 | 156 | 2.6 | 0.80 [0.63 – 1.01] | 0.82 [0.59 – 1.12] |
| ICS former users | 1,733 | 44 | 2.5 | 0.79 [0.56 – 1.11] | 0.77 [0.53 – 1.12] |
| **Mortality within 30 days after admission** | | | | | |
|  | **All patients** | **No. of Deaths** | **Mortality %** | **Crude RR**  **(95% CI)** | **Adjusted RR (95% CI)** |
| ICS non-users | 3,563 | 448 | 12.6 | 1 (referent) | 1 (referent) |
| ICS current users | 6,078 | 554 | 9.1 | 0.73 [0.64 – 0.82] | 0.72 [0.62 – 0.85] |
| Short-term ICS users | 745 | 72 | 9.7 | 0.77 [0.61 – 0.97] | 0.78 [0.61 – 1.00] |
| Longer-term ICS users | 5,328 | 482 | 9.0 | 0.72 [0.64 – 0.81] | 0. 71 [0.61 – 0.84 |
| ICS former users | 1,734 | 209 | 12.0 | 0.96 [0.82 – 1.12] | 0.89 [0.75 – 1.05] |
| **Intensive care unit admission** | | | | | |
|  | **All patients** | **No. of Events** | **%** | **Crude RR**  **(95% CI)** | **Adjusted RR (95% CI)** |
| ICS non-users | 1,597 | 142 | 8.9 | 1 (referent) | 1 (referent) |
| ICS current users | 3,624 | 207 | 5.7 | 0.64 [0.52 – 0.79] | 0.77 [0.57 – 1.04] |
| ICS former users | 944 | 54 | 5.7 | 0.64 [0.48 – 0.87] | 0.81 [0.58 – 1.13] |

Abbreviations: CAP = Community-acquired pneumonia; COPD = Chronic obstructive pulmonary disease; ICS = Inhaled corticosteroids; RR = Risk ratio; CI = Confidence interval

ICS current users: persons who had filled their most recent prescription within 180 days prior to the index date. Short-term ICS users: persons who had filled their first-ever prescription within 365 days prior to the index date. Longer-term ICS users: persons who had filled their first-ever prescription more than 365 days prior to the index date. Former users: persons who had redeemed their most recent prescription ≥ 181 days before the index date.

Non-users: persons with no redeemed ICS prescriptions ever before the index date.

Risk ratios for pleuropulmonary complications, 30-day mortality, and intensive care unit admission were adjusted for sex, age, myocardial infarction, congestive heart failure, cerebrovascular disease, diabetes, renal disease, liver disease, cancer, history of alcoholism-related disorders, COPD duration, status as a patient with frequent exacerbations, and pre-admission use of antibiotics, non-steroidal anti-inflammatory drugs, paracetamol, systemic steroids, immunosuppressants, inhaled bronchodilators, cardiometabolic drugs, proton pump drugs, and diabetes drugs.

## Table S6. Stratified analysis of the pleuropulmonary complication rate according to ICS use, by sex, age, CCI score, COPD duration, exacerbation history, ICS types, mean DDD, long-acting bronchodilator use, and study period.

|  | **All patients** | **No. of Events** | **%** | **Crude RR**  **(95% CI)** | **Adjusted RR**  **(95% CI)** |
| --- | --- | --- | --- | --- | --- |
| **Female*** | | | | | |
| ICS non-users | 1,560 | 44 | 2.8 | 1 (referent) | 1 (referent) |
| ICS current users | 3,075 | 71 | 2.3 | 0.82 [0.56 – 1.19] | Not convergent |
| ICS former users | 810 | 19 | 2.3 | 0.83 [0.49 – 1.41] | Not convergent |
| **Male*** | | | | | |
| ICS non-users | 2,002 | 71 | 3.5 | 1 (referent) | 1 (referent) |
| ICS current users | 2,998 | 85 | 2.84 | 0.80 [0.59 – 1.09] | 0.91 [0.60 – 1.38] |
| ICS former users | 923 | 25 | 2.71 | 0.76 [0.49 – 1.20] | 0.79 [0.49 – 1.30] |
| **Middle-aged persons (40 – 59 years)*** | | | | | |
| ICS non-users | 357 | 22 | 6.2 | 1 (referent) | 1 (referent) |
| ICS current users | 627 | 26 | 4.1 | 0.67 [0.39 – 1.17] | Not convergent |
| ICS former users | 185 | 8 | 4.3 | 0.70 [0.32 – 1.55] | Not convergent |
| **Older persons (60 – 79 years)*** | | | | | |
| ICS non-users | 2,007 | 60 | 3.0 | 1 (referent) | 1 (referent) |
| ICS current users | 3,813 | 98 | 2.6 | 0.86 [0.63 – 1.18] | 0.92 [0.61 – 1.37] |
| ICS former users | 993 | 28 | 2.8 | 0.94 [0.61 – 1.47] | 0.95 [0.59 – 1.53] |
| **Oldest old (80+ years)*** | | | | | |
| ICS non-users | 1,198 | 33 | 2.7 | 1 (referent) | 1 (referent) |
| ICS current users | 1,633 | 32 | 2.0 | 0.71 [0.44 – 1.15] | Not convergent |
| ICS former users | 555 | 8 | 1.4 | 0.52 [0.24 – 1.13] | Not convergent |
| **Low modified CCI score (0)**# | | | | | |
| ICS non-users | 1,336 | 36 | 2.7 | 1 (referent) | 1 (referent) |
| ICS current users | 2,553 | 72 | 2.8 | 1.05 [0.71 – 1.55] | Not convergent |
| ICS former users | 591 | 11 | 1.9 | 0.69 [0.35 – 1.35] | Not convergent |
| **Medium modified CCI score (1 - 2)**# | | | | | |
| ICS non-users | 1,500 | 53 | 3.5 | 1 (referent) | 1 (referent) |
| ICS current users | 2,434 | 59 | 2.4 | 0.69 [0.48 – 0.99] | 0.70 [0.41 – 1.18] |
| ICS former users | 725 | 21 | 2.9 | 0.82 [0.50 – 1.35] | 0.83 [0.47 – 1.46] |
| **High modified CCI score (3+)**# | | | | | |
| ICS non-users | 726 | 26 | 3.6 | 1 (referent) | 1 (referent) |
| ICS current users | 1,086 | 25 | 2.3 | 0.64 [0.37 – 1.10] | 0.77 [0.39 – 1.52] |
| ICS former users | 417 | 12 | 2.9 | 0.80 [0.41 – 1.58] | 0.84 [0.40 – 1.76] |
| **Duration of COPD ≤ 1 year**# | | | | | |
| ICS non-users | 1,939 | 60 | 3.1 | 1 (referent) | 1 (referent) |
| ICS current users | 1,834 | 44 | 2.4 | 0.78 [0.53 – 1.14] | Not convergent |
| ICS former users | 513 | 15 | 2.9 | 0.94 [0.54 – 1.65] | Not convergent |
| **Duration of COPD > 1 year**# | | | | | |
| ICS non-users | 1,623 | 55 | 3.4 | 1 (referent) | 1 (referent) |
| ICS current users | 4,239 | 112 | 2.6 | 0.78 [0.57 – 1.07] | 0.71 [0.47 – 1.07] |
| ICS former users | 1,220 | 29 | 2.4 | 0.70 [0.45 – 1.09] | 0.63 [0.39 – 1.02] |
| **Frequent exacerbator*** | | | | | |
| ICS non-users | 227 | 8 | 3.5 | 1 (referent) | 1 (referent) |
| ICS current users | 1,369 | 28 | 2.0 | 0.58 [0.27 – 1.26] | Not convergent |
| ICS former users | 251 | 3 | 1.2 | 0.34 [0.09 – 1.26] | Not convergent |
| **Non-frequent exacerbator*** | | | | | |
| ICS non-users | 3,335 | 107 | 3.2 | 1 (referent) | 1 (referent) |
| ICS current users | 4,704 | 128 | 2.7 | 0.85 [0.66 – 1.09] | 0.83 [0.59 – 1.18] |
| ICS former users | 1,482 | 41 | 2.8 | 0.86 [0.60 – 1.23] | 0.83 [0.56 – 1.24] |
| **Budesonide*** | | | | | |
| ICS non-users | 3,562 | 115 | 3.2 | 1 (referent) | 1 (referent) |
| ICS current users | 2,161 | 52 | 2.4 | 0.75 [0.54 – 1.03] | 0.89 [0.58 – 1.37] |
| ICS former users | 467 | 19 | 4.1 | 1.26 [0.78 – 2.03] | 1.32 [0.79 – 2.22] |
| **Fluticasone*** | | | | | |
| ICS non-users | 3,562 | 115 | 3.2 | 1 (referent) | 1 (referent) |
| ICS current users | 1,172 | 30 | 2.6 | 0.79 [0.53 – 1.18] | 0.80 [0.49 – 1.31] |
| ICS former users | 160 | 2 | 1.2 | 0.39 [0.10 – 1.55] | 0.39 [0.10 – 1.58] |
| **Beclometasone*** | | | | | |
| ICS non-users | 3,562 | 115 | 3.2 | 1 (referent) | 1 (referent) |
| ICS current users | 74 | 1 | 1.3 | 0.42 [0.06 – 2.96] | 0.50 [0.07 – 3.74] |
| ICS former users | 34 | 2 | 5.9 | 1.82 [0.47 – 7.07] | 1.88 [0.49 – 7.14] |
| **Mean**  **defined daily dose** # | | | | | |
| ICS non-users | 3,562 | 115 | 3.2 | 1 (referent) | 1 (referent) |
| ICS current users |  |  |  |  |  |
| Low dose (≤ 0.5 DDD) | 1,355 | 34 | 2.5 | 0.78 [0.53 – 1.13] | 0.76 [0.49 – 1.19] |
| Medium dose (0.5 – 1 DDD) | 1,772 | 52 | 2.9 | 0.91 [0.66 – 1.25] | 0.93 [0.62 – 1.38] |
| High dose (>1DDD) | 2,946 | 70 | 2.4 | 0.74 [0.55 – 0.99] | 0.72 [0.49 – 1.05] |
| **No long-acting bronchodilator*** | | | | | |
| ICS non-users | 3,152 | 98 | 3,1 | 1 (referent) | 1 (referent) |
| ICS current users | 1,459 | 32 | 2.2 | 0.71 [0.48 – 1.05] | 0.81 [0.52 – 1.25] |
| ICS former users | 1,028 | 28 | 2.7 | 0.81 [0.55 – 1.21] | 0.88 [0.57 – 1.37] |
| **LAMA only or LABA only*** | | | | | |
| ICS non-users | 368 | 16 | 4.4 | 1 (referent) | 1 (referent) |
| ICS current users | 2,551 | 57 | 2.2 | 0.51 [0.30 – 0.89] | 0.53 [0.30 – 0.94] |
| ICS former users | 544 | 11 | 2.0 | 0.47 [0.22 – 0.99] | 0.48 [0.22 – 1.05] |
| **LAMA & LABA*** | | | | | |
| ICS non-users | 42 | 1 | 2.38 | 1 (referent) | 1 (referent) |
| ICS current users | 2,063 | 67 | 3.25 | 1.36 [0.19 – 9.59] | Not convergent |
| ICS former users | 161 | 5 | 3.11 | 1.30 [0.16 – 10.87] | Not convergent |
| **1997-2004*** | | | | | |
| ICS non-users | 1,965 | 35 | 1.8 | 1 (referent) | 1 (referent) |
| ICS current users | 2,449 | 37 | 1.5 | 0.85 [0.54 – 1.34] | Not convergent |
| ICS former users | 789 | 18 | 2.3 | 1.28 [0.73 – 2.25] | Not convergent |
| **2005-2013*** | | | | | |
| ICS non-users | 1,597 | 80 | 5.0 | 1 (referent) | 1 (referent) |
| ICS current users | 3,624 | 119 | 3.3 | 0.66 [0.50 – 0.86] | 0.72 [0.48 – 1.09] |
| ICS former users | 944 | 26 | 2.8 | 0.55 [0.36 – 0.85] | 0.85 [0.35 – 0.93] |

Abbreviations: ICS = Inhaled corticosteroids; COPD = Chronic obstructive pulmonary disease; RR = Risk ratio; CI = Confidence interval; CCI = Charlson comorbidity index; DDD = Defined daily dose; LABA = Long-acting beta agonists; LAMA= Long-acting muscarinic antagonists

ICS current users: persons who had filled their most recent prescription within 180 days prior to the index date. Former users: persons who had redeemed their most recent prescription ≥ 181 days before the index date.

Non-users: persons with no redeemed ICS prescriptions before the index date.

* Risk ratios for pleuropulmonary complications were adjusted for sex, age, modified CCI score, history of alcoholism-related disorders, COPD duration, and pre-admission use of antibiotics, non-steroidal anti-inflammatory drugs, paracetamol, systemic steroids or immunosuppressants, inhaled bronchodilators, and cardiometabolic drugs.

# Risk ratios for pleuropulmonary complications were adjusted for sex, age, myocardial infarction, congestive heart failure, cerebrovascular disease, diabetes, renal disease, liver disease, cancer, history of alcoholism-related disorders, COPD duration, status as a patient with frequent exacerbations, and pre-admission use of antibiotics, non-steroidal anti-inflammatory drugs, paracetamol, systemic steroids, immunosuppressants, inhaled bronchodilators, cardiometabolic drugs, proton pump drugs, and diabetes drugs.

## Table S7. Stratified analysis of the 30-day mortality risk ratio according to ICS use, by sex, age, CCI score, COPD duration, exacerbation history, ICS types, mean DDD, long-acting bronchodilator use, and study period.

|  | **All patients** | **No. of Events** | **%** | **Crude MRR**  **(95% CI)** | **Adjusted MRR**  **(95% CI)** |
| --- | --- | --- | --- | --- | --- |
| **Female*** | | | | | |
| ICS non-users | 1,560 | 171 | 11.0 | 1 (referent) | 1 (referent) |
| ICS current users | 3,075 | 266 | 8.6 | 0.79 [0.66 – 0.95] | 0.75 [0.59 – 0.95] |
| ICS former users | 810 | 85 | 10.5 | 0.96 [0.75 – 1.22] | 0.84 [0.64 – 1.09] |
| **Male*** | | | | | |
| ICS non-users | 2,002 | 277 | 13.8 | 1 (referent) | 1 (referent) |
| ICS current users | 2,998 | 288 | 9.6 | 0.69 [0.59 – 0.81] | 0.71 [0.57 – 0.87] |
| ICS former users | 923 | 124 | 13.4 | 0.97 [0.80 – 1.18] | 0.93 [0.75 – 1.16] |
| **Middle-aged persons (40 – 59 years)*** | | | | | |
| ICS non-users | 357 | 20 | 5.6 | 1 (referent) | 1 (referent) |
| ICS current users | 627 | 17 | 2.7 | 0.48 [0.26 – 0.91] | 0.65 [0.29 – 1.46] |
| ICS former users | 185 | 10 | 5.4 | 0.96 [0.46 – 2.02] | 1.08 [0.52 – 2.25] |
| **Older persons (60 – 79 years)*** | | | | | |
| ICS non-users | 2,007 | 211 | 10.5 | 1 (referent) | 1 (referent) |
| ICS current users | 3,813 | 282 | 7.4 | 0.70 [0.59 – 0.83] | 0.67 [0.53 – 0.84] |
| ICS former users | 993 | 101 | 10.2 | 0.97 [0.77 – 1.21] | 0.87 [0.68 – 1.11] |
| **Oldest old (80+ years)*** | | | | | |
| ICS non-users | 1,198 | 217 | 18.1 | 1 (referent) | 1 (referent) |
| ICS non-users | 1,633 | 255 | 15.6 | 0.86 [0.73 – 1.02] | 0.80 [0.64 – 0.99] |
| ICS recent users | 555 | 98 | 17.7 | 0.97 [0.79 – 1.21] | 0.91 [0.72 – 1.16] |
| **Low modified CCI score (0)^#^** | | | | | |
| ICS non-users | 1,336 | 138 | 10.3 | 1 (referent) | 1 (referent) |
| ICS current users | 2,553 | 174 | 6.8 | 0.66 [0.53 – 0.82] | 0.61 [0.46 – 0.80] |
| ICS former users | 591 | 49 | 8.3 | 0.80 [0.59 – 1.10] | 0.76 [0.55 – 1.05] |
| **Medium modified CCI score (1 - 2)^#^** | | | | | |
| ICS non-users | 1,500 | 186 | 12.4 | 1 (referent) | 1 (referent) |
| ICS current users | 2,434 | 226 | 9.3 | 0.75 [0.62 – 0.90] | 0.79 [0.61 – 1.02] |
| ICS former users | 725 | 81 | 11.2 | 0.90 [0.70 – 1.15] | 0.86 [0.66 – 1.13] |
| **High modified CCI score (3+)^#^** | | | | | |
| ICS non-users | 726 | 124 | 17.1 | 1 (referent) | 1 (referent) |
| ICS current users | 1,086 | 154 | 14.2 | 0.83 [0.67 – 1.03] | 0.83 [0.62 – 1.10] |
| ICS former users | 417 | 79 | 18.9 | 1.11 [0.86 – 1.43] | 1.10 [0.82 – 1.46] |
| **Duration of COPD ≤ 1 year^#^** | | | | | |
| ICS non-users | 1,939 | 199 | 10.3 | 1 (referent) | 1 (referent) |
| ICS current users | 1,834 | 170 | 9.3 | 0.90 [0.74 – 1.10] | 0.91 [0.70 – 1.18] |
| ICS former users | 513 | 53 | 10.3 | 1.01 [0.76 – 1.34] | 0.98 [0.72 – 1.33] |
| **Duration of COPD > 1 year^#^** | | | | | |
| ICS non-users | 1,623 | 249 | 15.3 | 1 (referent) | 1 (referent) |
| ICS current users | 4,239 | 384 | 9.1 | 0.59 [0.51 – 0.69] | 0.63 [0.52 – 0.76] |
| ICS former users | 1,220 | 156 | 12.8 | 0.83 [0.69 – 1.00] | 0.82 [0.67 – 1.00] |
| **Frequent exacerbator*** | | | | | |
| ICS non-users | 227 | 41 | 18.1 | 1 (referent) | 1 (referent) |
| ICS current users | 1,369 | 126 | 9.2 | 0.51 [0.37 – 0.70] | 0.58 [0.39 – 0.86] |
| ICS former users | 251 | 41 | 16.3 | 0.90 [0.61 – 1.34] | 0.95 [0.62 – 1.44] |
| **Non-frequent exacerbator*** | | | | | |
| ICS non-users | 3,335 | 407 | 12.2 | 1 (referent) | 1 (referent) |
| ICS current users | 4,704 | 428 | 9.1 | 0.75 [0.66 – 0.85] | 0.80 [0.68 – 0.95] |
| ICS former users | 1,482 | 168 | 11.3 | 0.93 [0.78 – 1.10] | 0.90 [0.75 – 1.08] |
| **Budesonide*** | | | | | |
| ICS non-users | 3,562 | 448 | 12.6 | 1 (referent) | 1 (referent) |
| ICS current users | 2,161 | 192 | 8.9 | 0.71 [0.60 – 0.83] | 0.65 [0.53 – 0.80] |
| ICS former users | 467 | 51 | 10.9 | 0.87 [0.66 – 1.14] | 0.81 [0.61 – 1.07] |
| **Fluticasone*** | | | | | |
| ICS non-users | 3,562 | 448 | 12.6 | 1 (referent) | 1 (referent) |
| ICS current users | 1,172 | 98 | 8.4 | 0.66 [0.54 – 0.82] | 0.61 [0.47 – 0.78] |
| ICS former users | 160 | 19 | 11.9 | 0.94 [0.61 – 1.45] | 0.85 [0.55 – 1.33] |
| **Beclometasone*** | | | | | |
| ICS non-users | 3,562 | 448 | 12.6 | 1 (referent) | 1 (referent) |
| ICS current users | 74 | 6 | 8.1 | 0.64 [0.30 – 1.39] | 0.63 [0.29 – 1.38] |
| ICS former users | 34 | 8 | 23.5 | 1.87 [1.01 – 3.45] | 1.74 [0.94 – 3.24] |
| **Mean**  **defined daily dose ^#^** | | | | | |
| ICS non-users | 3,562 | 448 | 12.6 | 1 (referent) | 1 (referent) |
| ICS current users |  |  |  |  |  |
| Low dose (≤ 0.5 DDD) | 1,355 | 137 | 10.1 | 0.80 [0.67 – 0.96] | 0.80 [0.65 – 0.99] |
| Medium dose (0.5 – 1 DDD) | 1,722 | 156 | 8.8 | 0.70 [0.59 – 0.83] | 0.69 [0.56 – 0.85] |
| High dose (>1 DDD) | 2,946 | 261 | 8.9 | 0.70 [0.61 – 0.81] | 0.72 [0.59 – 0.87] |
| No long-acting bronchodilator* | | | | | |
| ICS non-users | 3,152 | 399 | 12.7 | 1 (referent) | 1 (referent) |
| ICS current users | 1,459 | 165 | 11.3 | 0.89 [0.75 – 1.06] | 0.83 [0.68 – 1.01] |
| ICS former users | 1,028 | 121 | 11.8 | 0.93 [0.77 – 1.13] | 0.81 [0.66 – 1.00] |
| LAMA only or LABA only* | | | | | |
| ICS non-users | 368 | 43 | 11.7 | 1 (referent) | 1 (referent) |
| ICS current users | 2,551 | 208 | 8.2 | 0.70 [0.51 – 0.95] | 0.73 [0.53 – 1.00] |
| ICS former users | 544 | 66 | 12.1 | 1.04 [0.72 – 1.49] | 1.03 [0.72 – 1.47] |
| LAMA & LABA* | | | | | |
| ICS non-users | 42 | 6 | 14.3 | 1 (referent) | 1 (referent) |
| ICS current users | 2,063 | 181 | 8.8 | 0.61 [0.29 – 1.31] | 0.71 [0.34 – 1.45] |
| ICS former users | 161 | 22 | 13.7 | 0.96 [0.41 – 2.21] | 1.01 [0.45 – 2.25] |
| **1997-2004*** | | | | | |
| ICS non-users | 1,965 | 249 | 12.7 | 1 (referent) | 1 (referent) |
| ICS current users | 2,449 | 225 | 9.2 | 0.73 [0.61 – 0.86] | 0.76 [0.61 – 0.93] |
| ICS former users | 789 | 101 | 12.8 | 1.01 [0.81 – 1.25] | 0.95 [0.76 – 1.20] |
| **2005-2013*** | | | | | |
| ICS non-users | 1,597 | 199 | 12.5 | 1 (referent) | 1 (referent) |
| ICS current users | 3,624 | 329 | 9.1 | 0.73 [0.62 – 0.86] | 0.68 [0.53 – 0.86] |
| ICS former users | 944 | 108 | 11.4 | 0.92 [0.74 – 1.14] | 0.82 [0.64 – 1.04] |

Abbreviations: ICS = Inhaled corticosteroids; COPD = Chronic obstructive pulmonary disease; RR = risk ratio; CI = Confidence interval; CCI = Charlson Comorbidity Index; DDD = Defined daily dose; LABA = Long-acting beta agonists ; LAMA= Long-acting muscarinic antagonists

ICS current users: persons who had filled their most recent prescription within 180 days prior to the index date. Former users: persons who had redeemed their most recent prescription ≥ 181 days before the index date.

Non-users: persons with no redeemed ICS prescriptions before the index date

* Risk ratios for 30-day mortality were adjusted for sex, age, modified CCI score, history of alcoholism-related disorders, COPD duration, and pre-admission use of antibiotics, non-steroidal anti-inflammatory drugs, paracetamol, systemic steroids or immunosuppressants, inhaled bronchodilators, and cardiometabolic drugs.

# Risk ratios for 30-day mortality were adjusted for sex, age, myocardial infarction, congestive heart failure, cerebrovascular disease, diabetes, renal disease, liver disease, cancer, history of alcoholism-related disorders, COPD duration, status as a patient experiencing frequent exacerbations, and pre-admission use of antibiotics, non-steroidal anti-inflammatory drugs, paracetamol, systemic steroids, immunosuppressants, inhaled bronchodilators, cardiometabolic drugs, proton pump drugs, and diabetes drugs.

## Table S8. Stratified analysis of the ICU admission rate according to ICS use, by sex, age, CCI score, COPD duration, exacerbation history, ICS types, mean DDD, and long-acting bronchodilator use.

|  | **All patients** | **No. of Events** | **%** | **Crude RR**  **(95% CI)** | **Adjusted RR**  **(95% CI)** |
| --- | --- | --- | --- | --- | --- |
| **Female*** | | | | | |
| ICS non-users | 687 | 61 | 8.9 | 1 (referent) | 1 (referent) |
| ICS current users | 1,862 | 117 | 6.3 | 0.71 [0.53 – 0.95] | 1.00 [0.66 – 1.52] |
| ICS former users | 466 | 29 | 6.2 | 0.70 [0.46 – 1.07] | 1.00 [0.63 – 1.57] |
| **Male*** | | | | | |
| ICS non-users | 910 | 81 | 8.9 | 1 (referent) | 1 (referent) |
| ICS current users | 1,762 | 90 | 5.1 | 0.57 [0.43 – 0.77] | Not convergent |
| ICS former users | 478 | 25 | 5.2 | 0.59 [0.38 – 0.91] | Not convergent |
| **Middle-aged persons (40 – 59 years)*** | | | | | |
| ICS non-users | 176 | 22 | 12.5 | 1 (referent) | 1 (referent) |
| ICS current users | 374 | 23 | 6.1 | 0.49 [0.28 – 0.86] | Not convergent |
| ICS former users | 111 | 6 | 5.4 | 0.43 [0.18 – 1.03] | Not convergent |
| **Older persons (60 – 79 years)*** | | | | | |
| ICS non-users | 799 | 77 | 9.6 | 1 (referent) | 1 (referent) |
| ICS current users | 2,134 | 143 | 6.7 | 0.70 [0.53 – 0.91] | 0.93 [0.61 – 1.42] |
| ICS former users | 341 | 35 | 7.1 | 0.74 [0.50 – 1.08] | 0.99 [0.63 – 1.56] |
| **Oldest old (80+ years)*** | | | | | |
| ICS non-users | 622 | 43 | 6.9 | 1 (referent) | 1 (referent) |
| ICS current users | 1,116 | 41 | 3.7 | 0.53 [0.35 – 0.81] | Not convergent |
| ICS former users | 341 | 13 | 3.8 | 0.55 [0.30 – 1.01] | Not convergent |
| **Low modified CCI score (0)**# | | | | | |
| ICS non-users | 549 | 58 | 10.6 | 1 (referent) | 1 (referent) |
| ICS current users | 1,363 | 95 | 7.0 | 0.66 [0.48 – 0.90] | 0.92 [0.57 – 1.50] |
| ICS former users | 277 | 17 | 6.1 | 0.58 [0.35 – 0.98] | 0.83 [0.48 – 1.47] |
| **Medium modified CCI score (1 - 2)**# | | | | | |
| ICS non-users | 642 | 54 | 8.4 | 1 (referent) | 1 (referent) |
| ICS current users | 1,478 | 80 | 5.4 | 0.64 [0.46 – 0.90] | 0.79 [0.49 – 1.25] |
| ICS former users | 391 | 25 | 6.4 | 0.76 [0.48 – 1.20] | 0.90 [0.54 – 1.49] |
| **High modified CCI score (3+)**# | | | | | |
| ICS non-users | 406 | 30 | 7.4 | 1 (referent) | 1 (referent) |
| ICS current users | 783 | 32 | 4.1 | 0.55 [0.34 – 0.90] | Not convergent |
| ICS former users | 276 | 12 | 4.3 | 0.59 [0.31 – 1.13] | Not convergent |
| **Duration of COPD ≤ 1 year**# | | | | | |
| ICS non-users | 877 | 100 | 11.4 | 1 (referent) | 1 (referent) |
| ICS current users | 963 | 68 | 7.1 | 0.62 [0.46 – 0.83] | 0.83 [0.56 – 1.23] |
| ICS former users | 253 | 17 | 6.7 | 0.59 [0.36 – 0.97] | 0.73 [0.44 – 1.24] |
| **Duration of COPD > 1 year**# | | | | | |
| ICS non-users | 720 | 42 | 5.8 | 1 (referent) | 1 (referent) |
| ICS current users | 2,661 | 139 | 5.2 | 0.90 [0.64 – 1.25] | 0.71 [0.43 – 1.16] |
| ICS former users | 691 | 37 | 5.3 | 0.92 [0.60 – 1.41] | 0.85 [0.52 – 1.40] |
| **Frequent exacerbator*** | | | | | |
| ICS non-users | 117 | 5 | 4.3 | 1 (referent) | 1 (referent) |
| ICS current users | 890 | 57 | 6.4 | 1.50 [0.61 – 3.66] | Not convergent |
| ICS former users | 123 | 7 | 5.7 | 1.33 [0.43 – 4.08] | Not convergent |
| **Non-frequent exacerbator*** | | | | | |
| ICS non-users | 1,480 | 137 | 9.3 | 1 (referent) | 1 (referent) |
| ICS current users | 2,734 | 150 | 5.5 | 0.59 [0.47 – 0.74] | 0.75 [0.55 – 1.03] |
| ICS former users | 821 | 47 | 5.7 | 0.62 [0.45 – 0.85] | 0.80 [0.56 – 1.14] |
| **Budesonide*** | | | | | |
| ICS non-users | 1,597 | 142 | 8.9 | 1 (referent) | 1 (referent) |
| ICS current users | 1,448 | 81 | 5.6 | 0.63 [0.48 – 0.82] | 0.81 [0.56 – 1.18] |
| ICS former users | 311 | 13 | 4.2 | 0.47 [0.27 – 0.82] | 0.59 [0.33 – 1.06] |
| **Fluticasone*** | | | | | |
| ICS non-users | 1,597 | 142 | 8.9 | 1 (referent) | 1 (referent) |
| ICS current users | 852 | 39 | 4.6 | 0.51 [0.36 – 0.73] | 0.73 [0.46 – 2.26] |
| ICS former users | 116 | 8 | 6.9 | 0.78 [0.39 – 1.54] | 1.19 [0.57 – 2.51] |
| **Beclometasone*** | | | | | |
| ICS non-users | 1,597 | 142 | 8.9 | 1 (referent) | 1 (referent) |
| ICS current users | 12 | 1 | 8.3 | Not convergent | Not convergent |
| ICS former users | 7 | 0 | 0 | Not convergent | Not convergent |
| **Mean**  **defined daily dose** # | | | | | |
| ICS non-users | 1,597 | 142 | 8.9 | 1 (referent) | 1 (referent) |
| ICS current users |  |  |  |  |  |
| Low dose (≤ 0.5 DDD) | 852 | 54 | 6.3 | 0.71 [0.53 – 0.96] | 0.84 [0.58 – 1.22] |
| Medium dose (0.5 – 1 DDD) | 1,067 | 50 | 4.7 | 0.53 [0.39 – 0.72] | 0.69 [0.46 – 1.02] |
| High dose (>1DDD) | 1,705 | 103 | 6.0 | 0.68 [0.53 – 0.87] | 0.87 [0.60 – 1.27] |
| No long-acting bronchodilator* | | | | | |
| ICS non-users | 1,314 | 121 | 9.2 | 1 (referent) | 1 (referent) |
| ICS current users | 362 | 25 | 6.9 | 0.75 [0.50 – 1.14] | 0.91 [0.57 – 1.45] |
| ICS former users | 473 | 27 | 5.7 | 0.62 [0.41 – 0.93] | 0.78 [0.50 – 1.22] |
| LAMA only or LABA only* | | | | | |
| ICS non-users | 234 | 16 | 6.8 | 1 (referent) | 1 (referent) |
| ICS current users | 1,393 | 76 | 5.5 | 0.80 [0.47 – 1.34] | Not convergent |
| ICS former users | 331 | 15 | 4.5 | 0.66 [0.33 – 1.31] | Not convergent |
| LAMA & LABA* | | | | | |
| ICS non-users | 37 | 4 | 10.8 | 1 (referent) | 1 (referent) |
| ICS current users | 1,845 | 105 | 5.7 | 0.53 [0.20 – 1.35] | 0.48 [0.19 – 1.26] |
| ICS former users | 135 | 12 | 8.9 | 0.82 [0.28 – 2.40] | 0.84 [0.29 – 2.43] |

Abbreviations: ICS = Inhaled corticosteroids; COPD = Chronic obstructive pulmonary disease; RR = Risk ratio; CI = Confidence interval; CCI = Charlson comorbidity index; DDD = Defined daily dose; LABA = Long-acting beta agonists ; LAMA= Long-acting muscarinic antagonists

ICS current users: persons who had filled their most recent prescription within 180 days prior to the index date. Former users: persons who had redeemed their most recent prescription ≥ 181 days before the index date.

Non-users: persons with no redeemed ICS prescriptions before the index date.

* Risk ratios for intensive care unit admission were adjusted for sex, age, modified CCI score, history of alcoholism-related disorders, COPD duration, and pre-admission use of antibiotics, non-steroidal anti-inflammatory drugs, paracetamol, systemic steroids or immunosuppressants, inhaled bronchodilators, and cardiometabolic drugs.

# Risk ratios for intensive care unit admission were adjusted for sex, age, myocardial infarction, congestive heart failure, cerebrovascular disease, diabetes, renal disease, liver disease, cancer, history of alcoholism-related disorders, COPD duration, status as a patient with frequent exacerbations and pre-admission use of antibiotics, non-steroidal anti-inflammatory drugs, paracetamol, systemic steroids, immunosuppressants, inhaled bronchodilators, cardiometabolic drugs, proton pump drugs, and diabetes drugs.

## Table S9. Pleuropulmonary complication, 30-day mortality, and intensive care unit admission rates after CAP admission, according to ICS use. Sensitivity analysis using a 90 days exposure window.

| **Pleuropulmonary complications** | | | | | |
| --- | --- | --- | --- | --- | --- |
|  | **All patients** | **No. of Events** | **%** | **Crude RR**  **(95% CI)** | **Adjusted RR**  **(95% CI)** |
| ICS non-users | 3,562 | 115 | 3.2 | 1 (referent) | 1 (referent) |
| ICS current users | 5,456 | 139 | 2.5 | 0.79 [0.62 – 1.01] | 0.80 [0.58 – 1.11] |
| ICS former users | 2,350 | 61 | 2.6 | 0.80 [0.59 – 1.09] | 0.79 [0.56 – 1.12] |
| **Mortality within 30 days after admission** | | | | | |
|  | **All patients** | **No. of Deaths** | **Mortality %** | **Crude RR**  **(95% CI)** | **Adjusted RR (95% CI)** |
| ICS non-users | 3,563 | 448 | 12.6 | 1 (referent) | 1 (referent) |
| ICS current users | 5,456 | 485 | 8.9 | 0.71 [0.63 – 0.80] | 0.71 [0.60 – 0.83] |
| ICS former users | 2,350 | 278 | 11.8 | 0.94 [0.82 – 1.08] | 0.88 [0.75 – 1.03] |
| **Intensive care unit admission** | | | | | |
|  | **All patients** | **No. of Events** | **%** | **Crude RR**  **(95% CI)** | **Adjusted RR (95% CI)** |
| ICS non-users | 1,597 | 142 | 8.9 | 1 (referent) | 1 (referent) |
| ICS current users | 3,286 | 182 | 5.5 | 0.62 [0.50 – 0.77] | 0.73 [0.55 – 0.99] |
| ICS former users | 1,282 | 79 | 6.2 | 0.69 [0.53 – 0.90] | 0.85 [0.63 – 1.16] |

Abbreviations: CAP = Community-acquired pneumonia; COPD = Chronic obstructive pulmonary disease; ICS = Inhaled corticosteroids; RR = Risk ratio; CI = Confidence interval

ICS current users: persons who had filled their most recent prescription within 90 days prior to the index date. Former users: persons who had redeemed their most recent prescription ≥ 91 days before the index date.

Non-users: persons with no redeemed ICS prescriptions before the index date.

Risk ratios for pleuropulmonary complications, 30-day mortality, and intensive care unit admission were adjusted for sex, age, myocardial infarction, congestive heart failure, cerebrovascular disease, diabetes, renal disease, liver disease, cancer, history of alcoholism-related disorders, COPD duration, status as a patient with frequent exacerbations, and pre-admission use of antibiotics, non-steroidal anti-inflammatory drugs, paracetamol, systemic steroids, immunosuppressants, inhaled bronchodilators, cardiometabolic drugs, proton pump drugs, and diabetes drugs.

## Table S10. 30-day mortality rate after CAP admission, according to ICS use. Sensitivity analysis using a Cox proportional-hazards model instead of Poisson model.

| **Mortality within 30 days after admission** | | | | | |
| --- | --- | --- | --- | --- | --- |
|  | **All patients** | **No. of Deaths** | **Mortality %** | **Crude HR**  **(95% CI)** | **Adjusted HR (95% CI)** |
| ICS non-users | 3,563 | 448 | 12.6 | 1 (referent) | 1 (referent) |
| ICS current users | 6,078 | 554 | 9.1 | 0.71 [0.63 – 0.81] | 0.72 [0.61 – 0.85] |
| ICS former users | 1,734 | 209 | 12.0 | 0.96 [0.82 – 1.13] | 0.91 [0.76 – 1.09] |

Abbreviations: CAP = Community-acquired pneumonia; COPD = Chronic obstructive pulmonary disease; ICS = Inhaled corticosteroids; HR = Hazard ratio; CI = Confidence interval

ICS current users: persons who had filled their most recent prescription within 180 days prior to the index date. Former users: persons who had redeemed their most recent prescription ≥ 181 days before the index date.

Non-users: persons with no redeemed ICS prescriptions before the index date.

Hazard ratios for 30-day mortality were adjusted for sex, age, myocardial infarction, congestive heart failure, cerebrovascular disease, diabetes, renal disease, liver disease, cancer, history of alcoholism-related disorders, COPD duration, status as a patient with frequent exacerbations, and pre-admission use of antibiotics, non-steroidal anti-inflammatory drugs, paracetamol, systemic steroids, immunosuppressants, inhaled bronchodilators, cardiometabolic drugs, proton pump drugs, and diabetes drugs.

## Table S11. Risk of all-cause mortality, respiratory mortality and cardiovascular mortality among a subcohort of 6,917 COPD patients followed from Jan 1, 2002, to Dec 31, 2011.

|  | **All-cause mortality** | | | **Respiratory mortality** | | | **Cardiovascular mortality** | |
| --- | --- | --- | --- | --- | --- | --- | --- | --- |
|  | **Crude HRs**  **(95% CI)** | **Adjusted HRs**  **(95% CI)** | **Crude HRs**  **(95% CI)** | | **Adjusted HRs**  **(95% CI)** | **Crude HRs**  **(95% CI)** | | **Adjusted HRs**  **(95% CI)** |
| ICS non-users | 1 (referent) | 1 (referent) | 1 (referent) | | 1 (referent) | 1 (referent) | | 1 (referent) |
| ICS current users | 0.71 [0.60 – 0.83] | 0.70 [0.56 – 0.87] | 0.72 [0.60 – 0.87] | | 0.71 [0.55 – 0.92] | 0.41 [0.29 – 0.58] | | 0.50 [0.32 – 0.80] |
| ICS former users | 0.88 [0.71 – 1.09] | 0.85 [0.67 – 1.08] | 0.93 [0.73 – 1.20] | | 0.90 [0.69 – 1.19] | 0.48 [0.29 – 0.79] | | 0.51 [0.29 – 0.87] |

Abbreviations: COPD = Chronic obstructive pulmonary disease; ICS = Inhaled corticosteroids; HR = Hazard ratio; CI = Confidence interval

ICS current users: persons who had filled their most recent prescription within 180 days prior to the index date. Former users: persons who had redeemed their most recent prescription ≥ 181 days before the index date.

Non-users: persons with no redeemed ICS prescriptions before the index date.

Hazard ratios for 30-day mortality were adjusted for sex, age, myocardial infarction, congestive heart failure, cerebrovascular disease, diabetes, renal disease, liver disease, cancer, history of alcoholism-related disorders, COPD duration, status as a patient with frequent exacerbations, and pre-admission use of antibiotics, non-steroidal anti-inflammatory drugs, paracetamol, systemic steroids, immunosuppressants, inhaled bronchodilators, cardiometabolic drugs, proton pump drugs, and diabetes drugs.
